# Cardiovascular Magnetic Resonance in Marfan syndrome

**DOI:** 10.1186/1532-429X-15-33

**Published:** 2013-04-15

**Authors:** Helen Dormand, Raad H Mohiaddin

**Affiliations:** 1 Manchester Heart Centre, Manchester Royal Infirmary, Oxford Road, Manchester, M13 9WL, UK; 2 Royal Brompton Hospital and National Heart & Lung Institute, Imperial College, Sydney Street, London SW3 6NP, UK

**Keywords:** Marfan syndrome, Aorta, CMR, CT, Echo

## Abstract

This review provides an overview of Marfan syndrome with an emphasis on cardiovascular complications and cardiovascular imaging. Both pre- and post-operative imaging is addressed with an explanation of surgical management. All relevant imaging modalities are discussed with a particular focus on cardiovascular MR.

## Introduction

Marfan syndrome (MFS) is a disorder of connective tissue structure and function, with a reported incidence of approximately 1 in 5000 individuals [1]. With no predilection for gender or ethnicity the UK prevalence is in the region of 10,000. In 1896, Professor Antoine Bernard-Jean Marfan, founder of paediatrics in France, described a 5 year old girl with disproportionately long limbs and digits and called the condition, dolichostenomelie (slender limbs). His subsequent work led to the eponymous naming of Marfan syndrome, (although this ‘index case’ is now felt to represent congenital contractural arachnodactyly, a phenotypically similar but separate condition) [1]. However, it was not until the 1930’s that the associated cardiovascular complications began to be recognised in the Western medical literature.

Other pathologies overlap clinically with Marfan syndrome. In ectopia lentis syndrome, lens dislocation occurs without the associated cardiac abnormalities; with familial thoracic aortic syndrome, aortic pathology exists without other features of MFS and Loeys-Dietz syndrome.

With our understanding of phenotypic variance, internationally agreed clinical criteria were defined, initially in 1986 (Berlin nosology) [2] and later revised in the Ghent nosology of 1996 [3]. A further revision of the guidelines proposed aortic root aneurysm and lens dislocation as cardinal clinical features [4], see Table 1 and 2 (A web based diagnostic tool for application of these criteria is available at http://www.marfan.org).

**Table 1:** Simplified revised Ghent criteria

**In the absence of a family history:**	**Diagnosis of MFS**	**In the presence of a family history:**	**Diagnosis of MFS**
Aorta (Z ≥ 2 or dissection) and ectopia lentis	Yes	Ectopia lentis	Yes
Aorta (Z ≥ 2 or dissection) and a causal FBN1 mutation	Yes	Systemic score ≥ 7	Yes
Aorta (Z ≥ 2 or dissection) and systemic features (≥7)	Yes	Aorta (Z ≥ 2 above 20 yr old, Z ≥ 3 below 20 yr, or dissection)	Yes
Ectopia lentis and a causal FBN1 mutation and aortic aneurysm	Yes		Yes

Z-score is derived from a measurement of the sinus of Valsalva indexed to body surface area.

Caveat: other conditions such as Loeys-Dietz syndrome must be excluded.

**Table 2:** Scoring of systemic features

**Feature**	**Score**
Wrist and thumb sign	3
Wrist or thumb sign	1
Pectus carinatum	2
Pectus excavatum/asymmetry	1
Hindfoot deformity	2
Plain pes planus	1
Pneumothorax	2
Dural ectasia	2
Protrusio acetabuli	2
Reduced upper/lower segment ratio and increased arm span/height and no severe scoliosis	1
Scoliosis or thoracolumbar kyphosis	1
Reduced elbow extension	1
Facial features (3/5)	1
Skin striae	1
Myopia > 3 diopters	1
Mitral valve prolapsed	1

Maximum total 20.

## Genetic basis and pathogenesis

MFS is an autosomal dominant condition exhibiting complete penetrance but variable expression. Up to one third of cases are thought to be spontaneous mutations, higher than previously thought [5,6]. Rarer recessive conditions displaying the MFS phenotype have also been described [7]. In the majority of cases a mutation in the fibrillin 1 (*FBN1*) gene located on chromosome 15 causes the fibrillinopathy [8]. The fibrillin glycoprotein was discovered in 1986 and linked in 1991 to the *FBN1* gene, chromosome 15 and MFS [9]. *FBN1* molecular analysis, now in clinical practice, provides valuable diagnostic information, particularly in children in whom aortic dilatation may not initially be present [10].

Histological abnormalities in the aortic wall were recognised by the 1950’s and descriptions can be found of disrupted elastic lamella; disorganised and hypertrophied smooth muscle fibres with increased vascular channels from the media to adventitia [11]. Over time, refined histological study revealed degradation of the extracellular matrix referred to previously as ‘cystic medial necrosis’ [12].

**Figure 1:**
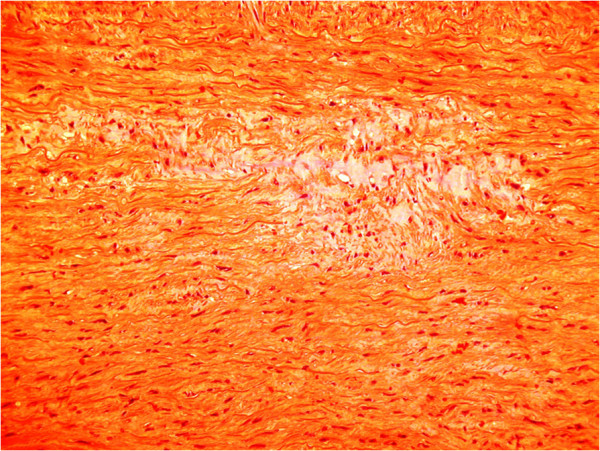
Haematoxylin and eosin stain showing cystic change in the vascular media in MFS.

**Figure 2:**
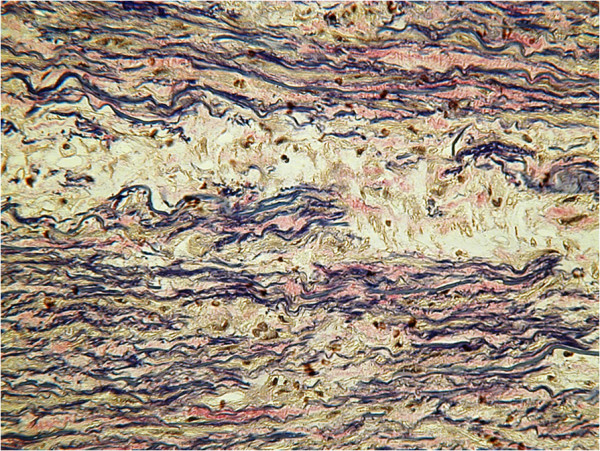
Trichrome stain where elastic fibres are black showing their destruction and fragmentation.

Fibrillin1 is a major component of 10-12 nm microfibrils which are an integral part of elastic connective tissues. They have complex structural, expansile and anchoring roles which are yet to be fully elucidated [9]. However, MFS is not merely a consequence of inherently weakened connective tissue as originally thought. Instead evidence from murine models now points towards an additional failure of appropriate maintenance of elastic fibres (Figures 1 and 2). Latent transforming growth factor-β (TGF-β) binding protein dysregulation by microfibrils is an important mechanism in MFS pathogenesis and can explain the phenotypic features found in many connective tissues [13,14]. The proteases involved in extracellular matrix degradation have also come under scrutiny and recent work has focused on matrix metalloproteinases and serine proteinases. These are implicated in all thoracic aneurysmal syndromes, not just MFS. In the aorta the defective tissues have increased alcianophilic glycosominoglycans, vacuoles secondary to the loss of smooth muscle cells and disordered adhesive protein. This renders them more susceptible to shear stress leading, over time, to dilatation and dissection.

## Systemic manifestations of MFS

The systems classically thought to be affected by MFS are cardiovascular, ocular and skeletal (see Table 1). Cardiovascular manifestations, particularly aortic dissection are the most common cause of death. Cardiovascular imaging is therefore fundamental to the screening, diagnosis and lifelong monitoring of these individuals.

**Figure 3:**
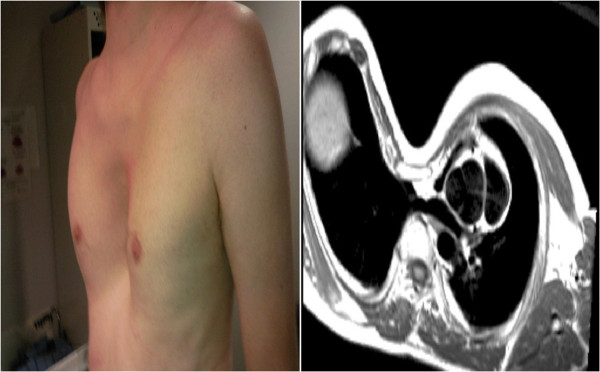
Pectus Excavatum in panel on the left with corresponding CMR image on the right.

**Figure 4:**
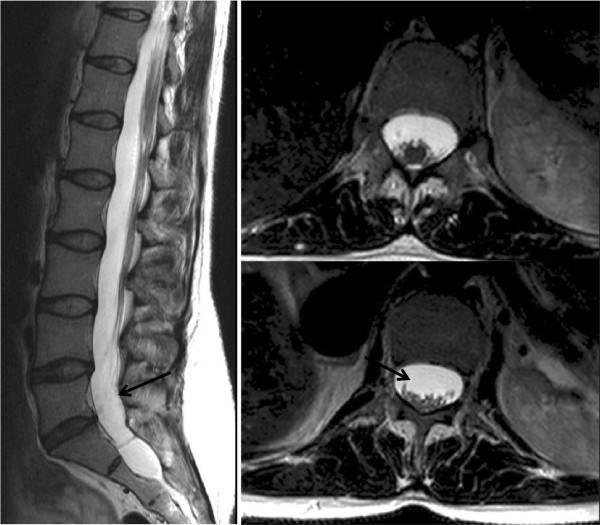
**CMR demonstrating dural ectasia. **This is ballooning or widening of the dural sac and has been the subject of various classifications. It may be associated with herniation of the nerve root sleeves through their associated foramina [134]. See arrows.

Upward subluxation of the lens (ectopia lentis) may be present in up to 80% patients; there is a tendency to myopia and an increased risk of retinal detachment [5]. Annual ophthalmological evaluation is advised. Although skeletal features may be most noted by clinicians, they feature less prominently in revised diagnostic guidelines and include dolichostenomelia (increased length of the limbs as compared with the trunk), arachnodactyly, scoliosis, pectus excavatum (Figure 3) or carinatum and dural ectasia (Figure 4). Pneumothorax and skin striae are also recognised systemic features.

## Phenocopies of MFS

The diagnosis of MFS is complicated by its overlapping phenotype with other conditions and is best made by an expert in this area, following internationally agreed criteria. For example, Professor Marfan’s index case is now thought to have had congenital contractural arachnodactyly which is related to mutations in the gene encoding for fibrillin 2 [12]. It is still fairly recently that Loeys-Dietz syndrome was recognised and is characterised by arterial tortuousity and aneurysms, hypertelorism, and bifid uvula or cleft palate. It is caused by heterozygous mutations in the genes encoding for transforming growth factor β receptors 1 and 2 (*TGFBR1, TGFBR2)*. Individuals with this autosomal dominant condition have aortic aneurysm formation and dissection at a much younger age than those with MFS and require annual MR of the vasculature from cerebrum to pelvis [15].

When mutations in fibrillin genes or *TGFBR1 or 2* are present without full phenotypic features of MFS or Loeys-Dietz, then individuals may be classified into any number of groups including ecoptia lentis syndrome and MASS syndrome (mitral, aortic, skeletal, skin), with some requiring imaging, particularly children in whom vascular features may present later and the diagnosis be changed. Type IV Ehlers-Danlos syndrome also predisposes to aortic dissection.

Significant numbers of thoracic aortic aneurysms are thought to follow an inherited pattern, even when no named syndromic cause can be identified, and screening for aortic and cerebral aneurysms in first degree relatives of affected individuals has been advocated [16].

## Treatment and prognosis in MFS

Medical therapy to reduce the rate of aortic dilatation and risk of dissection, once MFS has been diagnosed, is now advocated. Although experiments in turkeys over 50 years ago demonstrated that propranolol was effective in reducing death from dissecting aortic aneurysms [17], initial work in humans was not as successful [18]. Therefore it was not until 1994 that beta - blockade with propranolol was shown to be effective in slowing the rate of aortic dilatation and reducing the rate of complications in some people with MFS [19]. The beta-blocker should be titrated to effect, aimed at a heart rate after submaximal exercise of <100 beats/min in those over 5 years of age. Resting heart rate should be <60 beats/min if blood pressure will allow [6]. The rationale behind this therapy is one of reducing aortic wall shear stress by reducing the rate of pressure change in the aortic root and heart rate [20]. Subsequent work in a murine model of MFS demonstrated that losartan was effective in reducing aortic pathological changes and dilatation. Retrospective analysis in a group of paediatric patients with MFS appears to support this finding [21]. There is also evidence demonstrating that the ACE inhibitor perindopril reduces both aortic stiffness and aortic root diameter in MFS when taken in addition to beta-blockers [22]. Several randomised controlled trials are currently underway to evaluate the role of angiotensin receptor blockers and beta-blockers in MFS and should add considerably to our knowledge base.

If aortic dissection does occur then it constitutes a medical emergency. The majority of cases are Type A dissections usually necessitating surgery. Surgical treatment for aortic complications of Marfan syndrome continues to progress and will be discussed separately in the review. Lifelong imaging is recommended after aortic root surgery.

Exercise restriction may be recommended on an individual basis a part of the management of MFS and guidelines are provided by both the National Marfan Foundation (http://www.marfan.org) and the American Heart Association/American College of Cardiology task forces [23].

Earlier reports quoted a mean age at death of 32 years [24] and more recently 40 years [25]. This significant increase in life expectancy has been driven by medical and surgical intervention and the median cumulative probability of survival,(age at which half of a cohort would still be alive), has risen to around 70 years [26]. It has been recognised for some time that a family history of severe cardiovascular disease in MFS is associated with increased aortic diameter and decreased survival in individual patients [27].

## Cardiovascular complications

The significance of morphological cardiovascular manifestations in MFS was recognised early on [28]. They may be categorised as affecting the aorta, myocardium, valves or pulmonary arteries (see Table 3 for frequency). More recently the impact of skeletal abnormalities on cardiac function has been appreciated [29,30]. All need to be investigated by imaging.

**Table 3:** Frequency of cardiovascular involvement in adults with MFS

**Aortic root/Ascending aorta/Arch/Descending aorta**	**58**%**/19**%**/16**%**/15**%
Left ventricular impairment	25%
Mitral/Aortic Valves	80%/50%
Pulmonary Artery Dilatation	10%

For references see relevant section below.

### Aorta

In MFS, the aorta becomes the critical ground for the interplay between structural microfibril matrix abnormalities, heightened by failure of standard maintenance programmes by TGFβ, and beat to beat haemodynamic stressors. Endothelial shear stress, wall strain, torsion and intrinsic wall stress make this a dangerous environment for abnormal connective tissue. The result is a thinned aortic wall which progressively dilates and loses distensibility thereby heightening the risks of aneurysm formation and dissection throughout its length, but particularly at the root [6].

Cohort studies have shown that nearly 60% of those with MFS have root dilatation at a mean age of 35 years, with lower rates of more distal dilatation [31]. A review of patients with an FBN1 mutation, 73% of whom had MFS, demonstrated that the risk of ascending aortic (AA) dilatation increases with age, reaching 96% by the age of 60 years. Men were at higher risk than women for AA dilatation, dissection or surgery [32].

**Figure 5:**
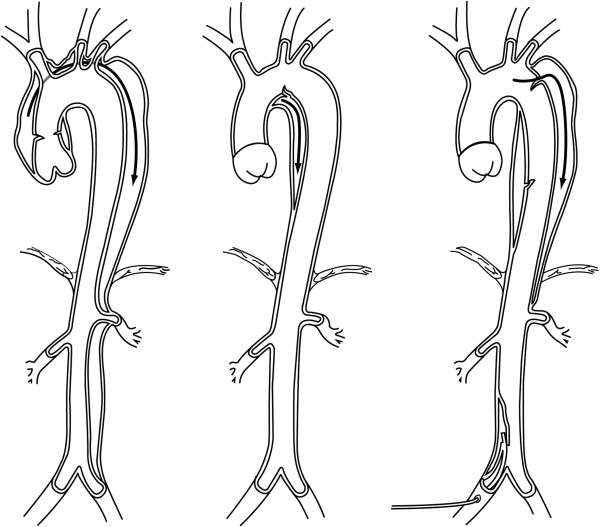
**Stanford and De Bakey classification of aortic dissection. **The image on the left represents De Bakey I and II/Stanford A; the middle image Stanford B; the right image De Bakey IIIa and b/Stanford B. De Bakey I; -origin in ascending aorta; extends to include at least arch; -usually surgical management. De Bakey II; -originates in and is confined to ascending aorta; -usually surgical management. De Bakey IIIa; -originates in descending aorta; usually extends distally; -usually nonsurgical management De Bakey IIIb; -originates in descending aorta; extends distally below diaphragm**; **-usually nonsurgical management. Stanford A; -all dissections involving ascending aorta; -management usually surgical. Stanford B; -all dissections not involving ascending aorta; -management usually nonsurgical.

Dissection is said to have occurred when the media is separated from the other aortic layers due to bleeding within and along the wall of the aorta. This is usually secondary to an intimal tear. It can be classified anatomically according to whether the ascending aorta is involved or by the site of the intimal tear, (Figure 5). This classification is important in clinical decision-making.

Dissection is acute if less than 2 weeks between onset of symptoms and presentation; subacute if 2 to 6 weeks gap; and chronic if more than 6 weeks has elapsed [33]. Similarly, the complications of dissection may also be acute, subacute or chronic.

Acute aortic dissection usually presents with pain, although around 6% of all dissections are painless but associated with increased mortality [34]. Approximately one quarter of those with untreated proximal dissections die within 24 hr of initial presentation. Death is usually from acute severe aortic regurgitation, aortic rupture or major branch vessel compromise.

Of those experiencing pain, it is usually midline and may be in the chest, back or abdomen depending on the location of the dissection. Onset is usually abrupt but only around half of patients describe the classical ‘tearing’ or ‘ripping’ quality so often highlighted at medical school. If this pain abates but then recurs it may indicate dissection extension and impending aortic rupture [35].

As extension occurs it may directly involve the walls of branch arteries. Alternatively branches may be compressed by expansion of a false lumen. Either mechanism leads to end organ compromise and additional systemic manifestations.

If the root is involved then aortic regurgitation may occur, as may pericardial effusion. Cardiac ischaemia may be the result of coronary artery involvement. However, regional wall motion abnormalities may occur as a result of generally low coronary perfusion pressures. Involvement of head and neck vessels can manifest as stroke or peripheral ischaemia. Peripheral ischaemia will lead to localising pain, pallor, paraesthesias and pulselessness of upper or lower limbs. Pulse deficit occurs in around 30% of patients and is associated with a poorer prognosis [36].

When renal ischaemia is present, there may be pain, haematuria, fever and biochemical abnormalities. The left renal artery is compromised more often than the right. If mesenteric ischaemia is present then there may be pain out of proportion to clinical findings and profound lactic acidosis. This is associated with a high mortality [37].

If aortic dissection is suspected on the basis of clinical assessment, then diagnostic imaging should be performed. Choice of imaging technique depends on patient stability, local expertise and availability. A second imaging modality should be performed if the first imaging is negative but clinical suspicion remains high.

**Figure 6:**
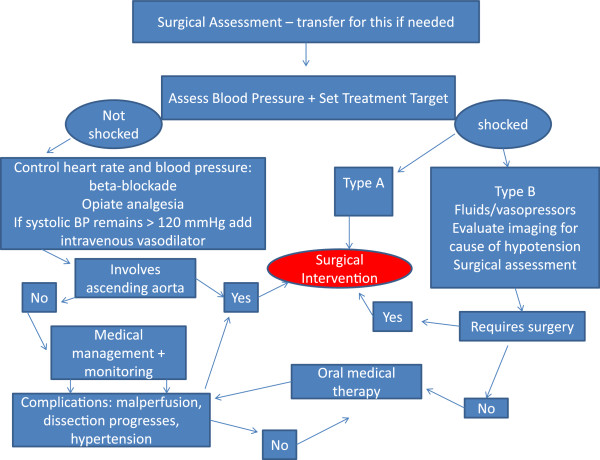
**Management pathway for acute aortic dissection. **Adapted from 2010 ACCF/AHA guidelines.

Once dissection is confirmed then initial management is aimed at limitation of propagation of the false lumen before a definitive management plan is instituted, (Figure 6).

In patients with MFS who have undergone aortic aneurysm repair, the ascending aorta is usually the site of the first operation, with mean age at time of surgery of 32.4 years. The majority have subsequent dissections or aneurysms, 95% of which are in other regions of the aorta. The presence of dissection at the time of first surgery is a significant predictor of requiring a second surgery [38]. In aortic dissection survivors with MFS, 80% have involvement of the ascending aorta, but about two thirds have involvement of the descending aorta or pure descending aortic dissection. Of those with dissection confined to the descending aorta, more than half have aortic root diameters less than 5 cm. Just over half of all patients incur a further clinical event during follow-up, usually in the descending aorta [39]. Of relevance, it has been shown that patients who have undergone elective aortic root surgery have a greater distal aortic diameter [40].

Aneurysms of the pulmonary trunk in MFS are rare [41], although dilatation of the main pulmonary artery is relatively common, occurring in over 10% patients [31,42].

### Valves

Aortic, mitral and tricuspid valves can be involved in MFS. The incidence of mitral valve prolapse and mitral regurgitation increases steadily with age, (although severe forms of MFS which may present in the neonatal period are associated with severe mitral and tricuspid disease) [43]. A survey of young patients with MFS (mean age 11.9 years) found over two thirds to have echocardiographic features of mitral valve dysfunction, usually prolapse. More than 25% of the cohort had disease progression over the following 4–6 years. However surgery for mitral regurgitation alone is uncommon in adults. The severity of mitral regurgitation in adults is unrelated to gender, unlike the risk of aortic dilatation and its complications [32].

The mitral valve changes associated with MFS are different to those associated with classic myxomatous mitral disease and are present in up to 80% adult patients. In MFS the leaflets are longer and thinner with an increased prevalence of bileaflet and anterior leaflet prolapse. However, posterior leaflet prolapse remains the most common valvular abnormality. In a surgical study patients with MFS were less likely to receive mitral valve repair than replacement. However, out of those receiving repair, the results were excellent with 96% freedom from reoperation at 10 years [44].

When the tricuspid valve is affected in MFS, it is usually in combination with the mitral valve. Similar to the mitral valve the pathologic mechanism of regurgitation is prolapse and the valve may be suitable for repair [45].

Aortic valve dysfunction is felt to be a later occurrence, usually secondary to annular dilatation [31]. However, the aortic valve is more than just a passive structure sitting between the left ventricular outflow tract and the aortic root. This is an important functional unit subject to complex haemodynamic forces. The sinuses of Valsalva have a particularly important role in the valve’s normal function.

As the aortic valve opens, the sinuses provide adequate space to prevent occlusion of the coronary artery orifices. In addition, this space permits the formation of eddy currents which hold the leaflets away from the aortic wall and facilitate appropriate valve closure at the end of systole. In diastole the sinuses move outwards and decrease the forces exerted on the valve leaflets.

As the aortic root dilates, the valve commissures will be moved outwards, thereby reducing leaflet coaptation in diastole, but also impairing the ability of the aortic sinuses and valve to function as one unit, thereby accelerating valvular dysfunction [46].

### Myocardial involvement

Fujiseki and colleagues reported the first case of MFS-associated myocardial involvement in 1984 [47]. Since then both right and left ventricular dysfunction and a dilated cardiomyopathy phenotype have been described [48]. Early studies using echocardiography found that a small proportion of patients had altered left ventricular dimensions [49]. Studies utilising more sensitive imaging techniques in those without valvular disease identified mild but significant impairment of left ventricular function [50]. Both systolic and diastolic impairment of function was identified by tissue doppler imaging (TDI). Importantly echocardiography was unable to identify any differences in left ventricular diameters, but CMR demonstrated a significant increase in end-systolic volume corrected for body surface area (BSA) and a decrease in ejection fraction. Left ventricular dilatation may predispose to alterations of repolarisation and fatal ventricular arrhythmias [51].

Biventricular involvement in MFS is well recognised. An echocardiographic study focusing on right ventricular systolic function found a reduction in the variables of TAPSE; rate of pressure rise (dp/dt) and peak TDI velocity of the basal lateral wall in those with MFS [52].

A comprehensive cardiac assessment was undertaken by Alpendurada et al. using cardiovascular magnetic resonance [53]. Evaluating patients with MFS and without cardiovascular surgery or significant valvular disease, 25% had a reduced left ventricular EF%. Just over 10% patients also had reduced right ventricular EF%. No patient with a normal LVEF% had a reduced RVEF%.

Although the degree of ventricular impairment in the vast majority of the patients featured in these imaging studies was mild, it is still early in the recognition of a ‘MFS-associated cardiomyopathy’. The natural history of this entity will be almost impossible to separate from developing aortic and valvular pathology in this patient group, but most authors recognise opportunities for pharmacologic intervention in these patients.

In addition to cardiomyopathic changes, the impact of skeletal abnormalities in MFS should also be considered. Recent studies have demonstrated that pectus excavatum can reduce resting right ventricular function [30]. Surgical correction of this deformity may improve cardiac function [29].

## Criteria for aortic surgical intervention

Just over a decade ago published surgical cohorts had a mean diameter of 6.8 cm, which was a reflection of both varied surgical practice but also of late or missed diagnosis of MFS in this patient population [54]. However the increasing incidence of a poor outcome after emergency surgery versus the low mortality of less than 2% for elective aortic replacement was already recognised and increasing numbers of surgeons were performing prophylactic root replacement at a diameter of 5.5 - 6.0 cm. Some groups in the late 1990’s were proposing a further reduction of this value to 5.0 - 5.5 cm [54-56].

American and European guidelines published in 2010 advocate surgical repair of a dilated aortic root/ascending aorta when the external diameter is 5.0 cm [33,57]. A summary and comparison of the guidelines is made in Table 4.

**Table 4:** Surgical repair of aortic root/ascending aorta recommended

**American guidelines**		**European guidelines**	
**External diameter > 5.0 cm**		**External diameter > 5.0 cm**	
Or at < 5.0 cm if:	Growth at greater 0.5 cm/yr	Or at 4.6-5.0 cm if:	Dilatation at greater 0.2 cm/yr
Or	Family history dissection at <5.0 cm	Or	Family history dissection
Or	Presence of significant aortic regurgitation	Or	Severe aortic or mitral regurgitation
Or in women contemplating pregnancy	>4.0 cm	Or	Pregnancy is being planned^#^
Also consider if:	Maximum CSA/height in metres >10 ^*^	In ‘small’ individuals use:	Indexed diameter adjusted for BSA of 2.75 cm/m^2^[58]

CSA = cross sectional area in cm^2 ^of ascending aorta or root BSA = body surface area.

^* ^Shorter patients have dissection at a smaller size. 15% patients with MFS have dissection at aortic root diameters less than 5.0 cm.

^#^Consider at 4.0-4.5 cm depending on serial growth on imaging and family history [59].

The American guidelines recognise the ongoing risk to the arch and descending aorta as sites for the later development of aneurysm formation and dissection, following prophylactic root repair. Routine lifelong imaging of the entire aorta is recommended. Whilst less data is available examining arch and descending aorta intervention, recommendations are made that in the presence of a chronic dissection, open repair of the descending thoracic aorta is undertaken at >5.5 cm. This is similar for the thoraco-abdominal aorta. Arch aneurysms are usually associated with disease of the adjacent proximal or distal aorta, and it is suggested that operative intervention in these individuals should be guided by the same parameters as for the adjacent segments.

European guidelines also give attention to the risk of dissection elsewhere and patients should be considered for surgery when other parts of the aorta are greater than 5.0 cm or progressive dilatation is evident [57].

## Surgical intervention

Surgery repairs or removes diseased tissue and structures, but alters the residual anatomy. Options for repair cover the spectrum from valve sparing root replacements to complete aortic valve, root and arch replacements, as well as other less invasive options. A basic understanding of the various surgical techniques is therefore important. This permits accurate and comprehensive reporting of pre and post operative images.

### The Bentall composite graft

**Figure 7:**
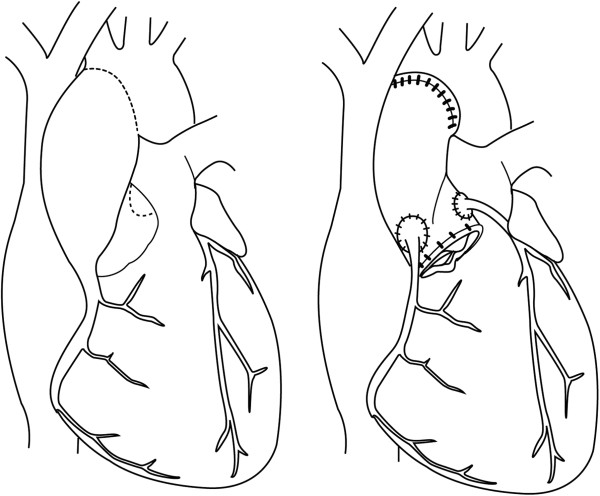
Bentall procedure.

The first published description of the original valved graft conduit repair of the aorta was in 1968 by Bentall (Figure 7) [60]. The technique was developed to allow treatment of those patients with aortic root aneurysm or dissection in whom the proximal aortic rim was insufficient to allow the suturing of a tubular graft and preservation of the native coronary ostial anatomy. Instead, the tubular graft was sutured directly onto the ring of a Starr valve and this was inserted en bloc. Holes were then cut in the aortic prosthesis to allow reinsertion of the coronary arteries.

Originally the graft material was unsealed and had to be pre-clotted outside the patient. This changed as pre-sealed material became available; then factory-made composite grafts and finally a revision of the coronary re-implantation technique with use of an aortic button [61]. As a consequence of increased operator experience and technical revisions the 30 day mortality from this operation fell to approximately 1.5% for elective cases [54,62]. However, rates for emergency repair remain significantly higher. 75% 10 year survival is reported, yet concerns persist with regard to long-term thromboembolic risks and the need for ongoing anticoagulation in such a young patient cohort who remain at life-long risk of further dissection. Some have therefore tried to use total root replacement surgery using tissue valves [63].

### Valve-sparing procedures

The aortic valve is a complex structure which cannot be matched by any prosthesis. The 3 cusps are more than passive sails, they interact with the root and LVOT. Aortic valve cusps are thin-walled pocket-like structures, yet strong and non-thrombogenic . The aortic root moves during cardiac cycle in a manner preceding and aiding opening and closing of AV [64-67]. Against this background valve-sparing root surgery was developed.

First Yacoub, then David proposed valve-sparing surgery [68,69] using variations of root replacement by an artificial graft with preservation of the patient’s native aortic valve.

**Figure 8:**
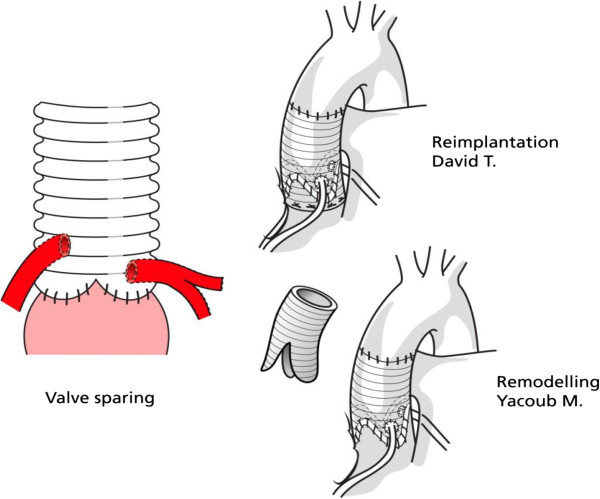
The David and Yacoub valve-sparing techniques for aortic root replacement.

In both, the sinuses of valsalva are resected first and a 3-4 mm rim of tissue to both sides of all commissures and along the attachment line of the cusps and coronary ostiae is left. The aortic root is replaced by a graft. The valve is then reimplanted into the vascular graft using a form of David technique or: can be remodelled into it as per the Yacoub technique (Figure 8).

In the Yacoub remodelling technique the graft first needs to be incised at its base so that the 3 commissures of the valve can be sewn into the 3 ‘tongues’ of the graft. Hence pseudo-sinuses are created within the vascular graft. The sinuses within the aortic root are considered important for aortic valve function and coronary perfusion. Finally the coronary ostiae have to be reimplanted into the graft.

In the original David re-implantation technique the vascular graft is placed over the native aortic valve and secured below the aortic valve attachment. Then the aortic valve and commissures are secured within the graft. As a result the valve sits within the tubular graft without the formation of pseudosinuses. Again the coronary ostiae have to be reimplanted.

The results of the original David procedure are generally considered to be better than with the Yacoub procedure in MFS. This may be related to the restricted movement and reduced distensibility caused by the original David procedure. It is also hypothesised that the graft incisions in the Yacoub procedure might limit the ability of the graft to reduce further annular dilatation in MFS [70]. This remains the case even with additional annuloplasty.

The David technique has been revised several times and forms of version V are currently in use [71]. This retains the implantation of the valve within the graft, but additionally allows the creation of Dacron pseudosinuses. Interestingly, time-resolved 3D MR velocity mapping can demonstrate formation of coronary cusp vortices post David repair, even in the absence of pseudosinus formation [72].

### Valve - sparing versus non valve-sparing surgery

A recent systematic review of the surgical management of aortic disease in MFS compared the results of total root replacement (TRR) versus valve-sparing aortic root replacement (VSRR) [73]. The longer established TRR had been performed in the majority with a mean follow-up of 8 years; for VSRR the figure was 4.7 years. VSRR was associated with a fourfold increased rate of reintervention on the aortic valve (1.3% per year vs 0.3%); with reintervention most likely in those undergoing remodelling of the root (Yacoub technique). On the other hand, TRR was associated with a significantly higher rate of thromboembolism (0.7% per year versus 0.3% per year). There was no difference between the two techniques in the composite valve-related event. However, durability data for VSRR are still lacking and further registry data are awaited.

Both the American and European guidelines advocate the use of valve-sparing operations with root replacement in cases in which the aortic valve is anatomically normal and in high-volume centres. Only if valve-sparing surgery is not possible then root replacement with a valved graft conduit is advocated. However, caution is expressed with regard to long-term durability of residual aortic valve function and in this context the American guidelines recommend a reimplantation technique rather than remodelling.

### External aortic root support

**Figure 9:**
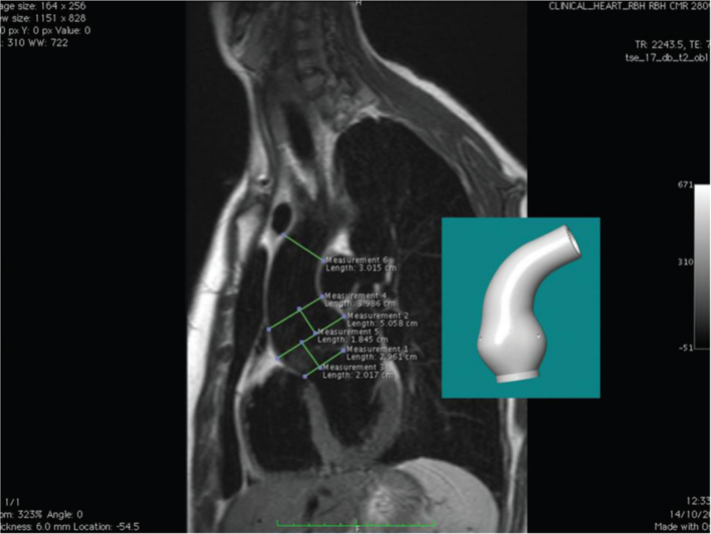
**Example of black blood aortic CMR imaging, some of the measurements and the model made by computer aided design. **Reproduced from the Journal Royal Society of Medicine 2010:103:370-375 [76].

**Figure 10:**
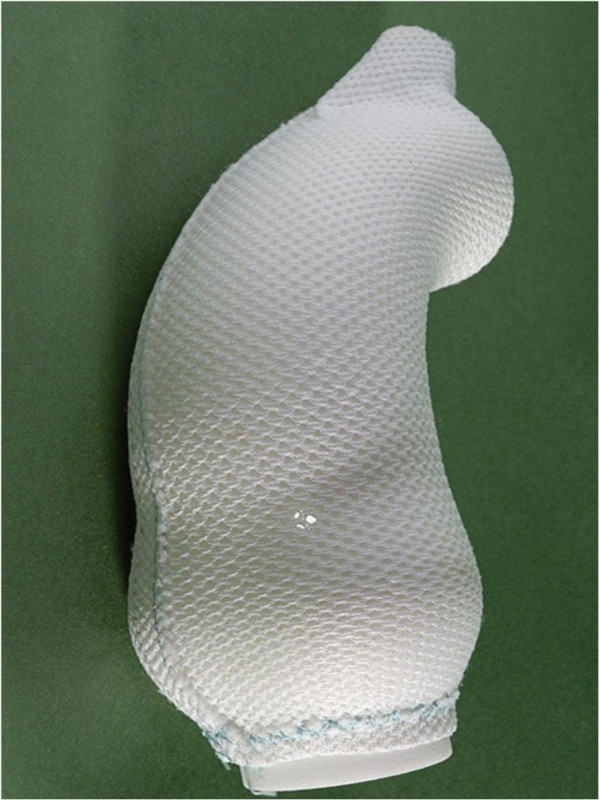
The aortic model covered with the external aortic root support made from a medical grade polymer mesh.

A further surgical technique has been pioneered and evaluated by NICE in 2011 [74]. An individualised external aortic root support (EARS) is used to reinforce the ascending aorta while leaving the native aortic valve intact and conserving the blood/endothelium interface [61]. Robicsek suggested many years ago that wrapping the aorta externally could prevent expansion. However the results of this early operation have not been good [75]. Furthermore, in the context of an already dilated aorta, the compromise of a hand-tailored external support at a time when “off the shelf” composite valve conduits were becoming available was worrying and not adopted in the main [54]. However, modern imaging and engineering methods have allowed us to revisit that idea. The recent development is born of the motivation, skill and creativity of a biomedical engineer with MFS who was the first individual to undergo this procedure just over 7 years ago. The clinical results for the first twenty patients were published in 2010 [76]. Each patient underwent CMR study of the aorta (more recently Cardiac CT has also been used) to provide digital information to produce a 3D reconstruction of the aorta from the aortoventricular junction to beyond the brachiocephalic artery using dedicated computer-aided design software. This was transformed into a thermoplastic model formed exactly to the physical model of the patient’s aorta. The latter was then used as the frame upon which the bespoke external aortic support was manufactured from a medical grade polymer mesh (Figures 9 and 10). At surgery, without the need for bypass, the aorta was dissected away from adjacent structures before being encircled by the external support. The external support was secured without any direct incision or suturing of aortic tissue. Whilst current follow-up data is very hopeful for long-term outcomes, should surgery be required on any part of the aorta in future in these patients, the avoidance of anticoagulation will be of benefit [77]. This approach has a number of potential advantages over standard treatment for Marfan Syndrome:

1. External support may reduce mechanical stress on the aortic wall, thus reducing repetitive mechanical injury due to the pulse wave and retarding the degenerative process.

2. The operation can be performed without the need for cardiopulmonary bypass, myocardial ischaemia or circulatory arrest.

3. It avoids replacing the aortic valve and therefore life-long anticoagulation

NICE recognise that at the present time the outcome data is based on a relatively small number of patients and further data with regards to long term outcomes is awaited. Hence this procedure is undertaken by specialist teams only with collection of audit and clinical data.

### Endovascular stent grafting

Endovascular stent grafting has been used in a few cases, however, long-term data are lacking and there are concerns about the ability of fragile aortic tissue to withstand the pressure of the stent over a long time. Such an approach should only be considered if the risks of open repair are prohibitive [57,78]. Aortic surgery has also been successfully combined with cardiac transplantation or left ventricular assist device implantation in patients with MFS [79,80].

## Cardiac imaging

With the ever growing appreciation of the complex manifestations of MFS, it has become apparent that patients need assessment of the entire aorta and careful study of valves and biventricular function [81]. As the number and quality of available imaging modalities has increased, it should have become ever easier to provide this imaging. However, a review of the literature reveals a heterogeneity of approaches and intermodality validation based on techniques or views which may not be routinely used. It is worth reviewing the current guidelines before reviewing each imaging modality individually, with an emphasis on cardiovascular MR (CMR).

## Imaging guidelines

The European guidelines recommend using 2 modalities in each patient and make recommendations for both diagnostic and follow-up imaging [57] (Table 5).

**Table 5:** European guidelines for imaging in MFS

**Diagnostic**	**Follow-up**
TTE of aortic root: measure at annulus, sinuses of Valsalva, sinotubular junction and distal ascending aorta.	TTE on an annual basis if stable
TTE of left ventricular and valvular function.	
Either CMR or CT of entire aorta *	CMR or CT of entire aorta: every 5 yr if normal aortic dimensions beyond root; at least annually if aneurysm formation beyond root.
**Avoid coronary angiography due to increased dissection risk**		
CT coronary angiography pre-op to assess for coronary disease where possible		
TOE- only in assessment of suspected aortic dissection		

*CMR assessment of aortic wall biophysical properties may be possible – see CMR section below.

American guidelines also recommend a TTE is performed at diagnosis to establish aortic dimensions, ventricular and valvular function, however, it should be repeated at 6 months to establish the rate of change of aortic parameters. If there is no significant change then annual TTE will suffice. If however there is significant aortic expansion or the initial aortic diameter is >4.5 cm, then more frequent imaging is advised. The guidelines recognise that most patients with MFS also undergo X-ray computed tomography (CT) or CMR but they do not give any specific guidance in this respect. They note that measurements made on these modalities are 2-4 mm greater than those on TTE, but it is on these external diameter measurements that surgical intervention is usually planned. TOE is again reserved for the setting of dissection.

Following surgical repair of the aortic root or ascending aorta, lifetime imaging of the entire aorta is recommended, in line with the European position.

Specific consideration is given to women with MFS during pregnancy and monthly or bimonthly echo is recommended in those with aortic root or ascending aortic dilatation. If the more distal aorta is affected then CMR without gadolinium may be used for monitoring during pregnancy.

The American guidelines address more than just the choice and timing of modality. They stress the importance of the comprehensive and reproducible nature of reporting of aortic disease which is key in MFS. An aneurysm is defined as: ‘a permanent localised dilatation of an artery, having at least a 50% increase compared with the expected normal diameter of the artery in question.’ The term ‘ectasia’ refers to lesser degrees of arterial dilatation.

**Figure 11:**
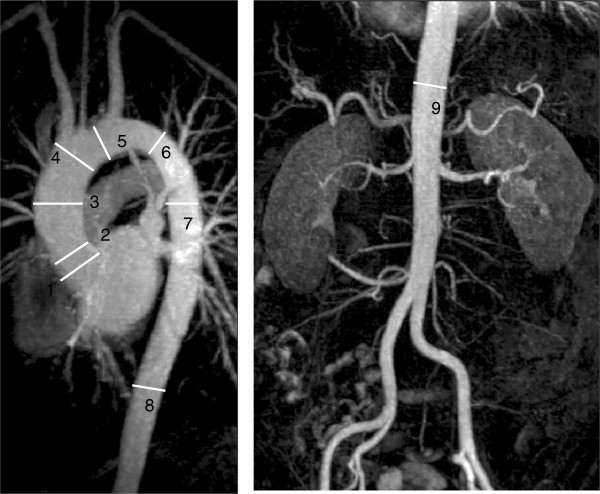
**CMR aorta demonstrating standard imaging landmarks for the thoracoabdominal aorta advocated by guidelines. **1: Aortic sinuses of Valsalva 2: Sinotubular junction 3: Mid ascending aorta 4: Proximal aortic arch (at origin of inominate artery) 5: Mid aortic arch (between left common carotid and subclavian arteries) 6: Proximal descending thoracic aorta (approx 2 cm distal to left subclavian artery) 7: Mid descending aorta 8: Aorta at diaphragm (2 cm above origin of coeliac axis) 9: Abdominal aorta at origin of coeliac axis.

Analysis of aortic images should be undertaken at a workstation which permits rotation of the acquired images to review each segment of the aorta and its branches. Reports should contain essential elements and measurements of aortic diameter should be taken at reproducible landmarks, perpendicular to the longitudinal or flow axis of the vessel (Figure 11). Diameter measurements from axial images are highlighted as being inherently incorrect unless properly aligned.

Recognition of the detailed analysis required to produce robust reports is welcomed by imagers. This is in keeping with SCMR guidance on analysis of thoracic magnetic resonance angiography and the multiple sequences which may be required for a comprehensive study of the aorta [82]. SCMR also recognises the need for timely reporting which will be influenced by clinical urgency, but ideally within 24 hours of a routine scan a finalised report should be available. As a minimum SCMR recommends measurement at the aortic annulus; sinuses of Valsalva, sinotubular junction and ascending and descending diameters at the level of the pulmonary artery. It is also recommended to report, when present: sinotubular effacement, tortuousity, aortic aneurysm and aortic dissection [83]. An aneurysm should be described in terms of size, morphology, location, mural thrombus, local effects on surrounding structures, post contrast appearance (if contrast given) and any pericardial, pleural, mediastinal or periaortic fluid. A similar level of detail is required for description of a dissection (either Stanford or De Bakey classification is acceptable) including the location of the tear or evident communication and whether an intimal flap is visible.

The American guidelines for CT and CMR recommend that the external aortic diameter should be measured and at the root the widest diameter, usually at mid-sinus level, should be taken. The latter also applies in echo but here the internal diameter should be measured [33]. However the rationale for this is that lumen size may not accurately reflect external diameter in the setting of mural thrombus, intramural haemtoma, thrombosed false aneurysm and aortic inflammation. Yet in the setting of normal aortic wall thickness, measuring external diameter will add a few millimeters (~3-4 mm) to the size of the aorta compared with the internal diameter measurements which may have impact on the timing of surgery/intervention. Furthermore, this will be at disparity with the echo measurement. In addition, most of the data and cutoff values used for surgical intervention are based on echo internal diameter measurements. Indeed, the guidelines state that Marfan aorta of more than 5 cm is at risk but avoids saying whether that’s internal or an external diameter.

In our centres we are still using internal aortic diameter measurements but are vigilant for any abnormal aortic wall pathology (e.g. intramural haematoma, dissection, ulceration, aortitis, etc.) and describe these lesions with measurements separately in the CMR/CT reports to address the guidelines’ concerns. We also have lots of patients who have been followed up for years using internal diameter measurements and as a group (imagers and surgeons), we felt that it is important to have continuity in these measurements.

In essence, to minimize errors, it is important that when following up patients for changes in aortic dimensions that measurements should be done at a reproducible anatomical plane perpendicular to the axis of flow and any abnormality of the aortic wall described and measured. When possible measurements should be indexed to body surface area.

Radiation exposure should be minimised wherever possible. Invasive angiography is no longer considered as a first-line investigation for aortic diseases and is rarely used in this setting.

## Imaging modalities

### Echocardiography

The portability, safety and cost-effectiveness of TTE have facilitated its fundamental role in both the diagnosis and monitoring of the aortic root in those with MFS. M-mode recordings were used to establish the effect of propranolol on the aortic root [19]. Recent studies have also used M-mode interchangeably with 2D-images to assess aortic root size [59]. Subsequent work has been undertaken which validates ‘inner edge to inner edge’ measurements of the proximal aorta against TOE [84] and TTE forms an integral part of most imaging guidelines in MFS. Yet a review of the literature demonstrates variability in the approach to TTE root measurement [85].

Yet the strength of TTE is that it may also assess valvular and biventricular function in both adults and children. As our understanding of MFS has grown, the assessment and monitoring of biventricular function per se should be part of our assessment, with tissue doppler imaging incorporated into any protocol used, although this has yet to feature explicitly in guidelines [86]. Chest wall deformities preclude adequate imaging in only the minority of patients with MFS.

**Figure 12:**
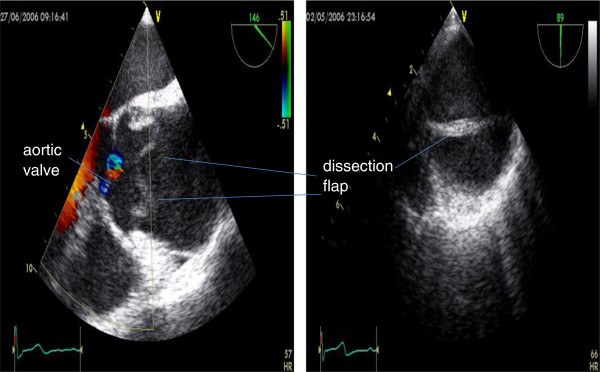
a) Dilated aortic root on transoesophageal echo with evidence of dissection flap b) Transaxial view of aortic dissection.

TOE is used mainly in the setting of suspected acute aortic root dissection in MFS. In experienced hands it has a sensitivity and specificity comparable to CT and CMR [87]. The echocardiographic diagnosis of dissection requires demonstration of a dissection flap separating true and false lumens. There may be more than one intimal tear present and colour Doppler may be used to show differential flow on either side of a flap. The true lumen usually expands during systole and collapses during diastole with forward flow during systole. A false lumen shows the reverse expansion pattern, accompanied by reversed, delayed or absent flow. Spontaneous contrast may be present. Assessment of aortic valve competency, presence of pericardial effusion and involvement of the coronary ostia may help guide surgical intervention. Increasingly intra-operative TOE is being used for assessment and monitoring. However, as a semi-invasive procedure which has the potential to raise a patient’s blood pressure, it is not used for routine aortic surveillance (Figure 12).

### X-ray computed tomography (CT)

CT is one of the longest serving imaging modalities in the assessment of aortic disease. It is a rapid test which can be used in either the acute or chronic disease setting to evaluate the entire aorta and periaortic structures. It can distinguish between acute aortic syndromes and elucidate branch vessel involvement. With the advent of ECG-gating, the risks of motion-artefact mimicking dissection have been greatly reduced. International Registry data have previously shown CT to be the first-line investigation in acute dissection in 61% cases [88]. Sensitivities of up to 100% and specificities of 92-100% for helical CT have been reported in this setting [87]. A non-contrast study to look for intramural haematoma, followed by a contrast study to identify a dissection flap and the contrast extravasation of rupture is recommended [33]. The vascular tree from neck to pelvis can be rapidly imaged to provide valuable information to the referring team and it is possible to obtain a CT coronary angiogram and aortogram in one ECG-gated CT acquisition. CT features which discriminate between the true and false lumen have been described [89]. Axial measurements tend to over-estimate the thoracic aortic diameter and measurements planned from double-oblique images are preferred [90]. Recommendations on technical parameters for acquisition and reconstruction are available [33].

Where used pre-operatively, cardiac CT offers the ability to perform a complete evaluation of the thorax. In patients with MFS this has the advantage of assessing the chest wall and surgical access with particular reference to coronary artery position, in those with chest wall deformities [91].

With the expansion in cardiac CT angiography, attempts have been made to evaluate its performance in valvular disease. A recent retrospective study comparing CT with TTE calculated a 96% sensitivity and 93% sensitivity for the diagnosis of mitral valve prolapse. However, it should be noted that this required retrospective gating to acquire the dataset and hence radiation exposure ranged from 7-11 mSv between patients. However, when retrospective gating has been required for other reasons, it may be worth looking at the dataset not only for MVP but also for an assessment of aortic valvular and left ventricular function. Currently there are limitations in the ability of CT to grade valvular dysfunction [92]. However on mid-systolic images the tethered appearance of the aortic valve resulting from sinus of Valsalva dilatation and a triangular coaptation defect may be present in end-diastole if aortic regurgitation is present [93].

The ability of CT to demonstrate and assess vasculature is not in doubt and, in the acute setting it can allow rapid diagnostic imaging assessment of an unwell patient. However, it is a technique involving the use of ionising radiation. Therefore it is not ideally suited for long term follow up, particularly in a young patient cohort whose lifetime accumulated dose could be considerable. ECG-gated low-voltage techniques significantly reduce ionising radiation exposure and minimise potential long-term harm [94].

### Aortography/angiography

Aortography was first used in the 1960s as a method for assessing aortic dissection. However it is invasive, requires the use of iodinated contrast and radiation. Whilst in the setting of primary PCI it is still possible to find oneself diagnosing acute aortic dissection by this method, it is otherwise rarely used and is not advocated in the setting of MFS.

## Cardiovascular magnetic resonance (CMR)

### Advantages and limitations

CMR is not limited by acoustic windows and is free from ionising radiation. The entire aorta can be imaged and complications including aneurysm formation, dissection, and previous surgery are well visualised. This makes it ideal for long-term follow up of patients.

Anatomical information can be gained, including origin and exit of intimal tears. CMR has equivalent sensitivity and specificity to CT for diagnosis of suspected thoracic aortic dissection, but is more accurate when the pre-test probability of dissection is high [87]. It has been validated against echocardiography for aortic root measurement and is well recognised as being superior at demonstrating the asymmetrical root dilatation which is often seen in MFS [95].

CMR allows assessment of both global and regional biventricular function and allows visualisation of the consequences of valvular dysfunction. Blood flow can be assessed in both normal and abnormal vessels. Reconstructions in 3 dimensions are a powerful tool for demonstrating anatomy to colleagues and patients alike. Utilising various sequences this technique is unsurpassed at characterising vascular and myocardial tissue.

The requirement for breath-holding limits the use of CMR in the unstable patient; however, availability and expertise are increasingly enabling studies to be tailored to the individual patient.

### Proposed CMR protocol

**Figure 13:**
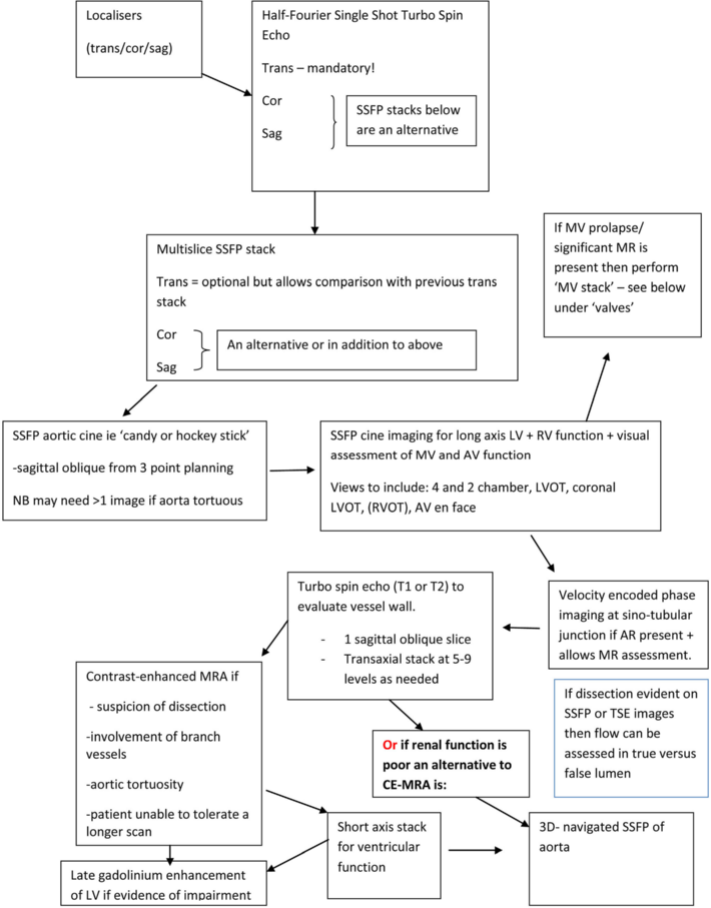
**CMR protocol. **Key to protocol abbreviations: trans = transaxial; cor = coronal; sag = sagittal; MV = mitral valve; AV = aortic valve; MRA = magnetic resonance angiography; TSE = turbo spin echo. Typical Imaging Parameters and points to note: Aortic SSFP- FoV Read 320 mm, slice thickness 7 mm, flip angle 80^0^, voxel size 1.7 × 1.7 × 7.0 mm**. **TSE – 5–10 parallel images planned from a transverse stack along long axis of aorta. Image position can be copied from Half-Fourier Single Shot TSE or SSFP stacks. For T2 weighted TSE: FoV Read 340 mm, slice thickness 6.0 mm, TR 700 ms, TE 81.0 ms**.** For T1 weighted TSE: FoV Read 340 mm, slice thickness 6.0 mm, TR 750 ms, TE 31.0 ms**. **CE-MRA- Give 0.1-0.2 mmol/kg gadolinium. Images should be reconstructed in MPR and analysed in thin MIP. 3D Navigated SSFP MRA- FoV Read 320 mm, slice thickness 1.5 mm, flip angle 90^0^, voxel size 1.0 × 1.0 × 1.5 mm.

We offer the following CMR protocol for imaging a patient with MFS in the pre-operative setting, (Figure 13).

### Protocol note: aortic distensibility assessment

High-resolution cine imaging in a plane perpendicular to the ascending and/or descending aorta allows measurement of aortic cross-sectional area during systole and diastole. Measurement of regional aortic distensibility by CMR is calculated from the change in volume of an aortic segment and from aortic pulse pressure estimated by a sphygmomanometer at the level of the brachial artery. The lumen of the aorta is outlined manually on the computer screen to measure the change in aortic area (*Δ*A) between diastole and systole. Aortic distensibility can be derived from the change in volume (*ΔV* = *ΔA* × slice thickness) of the aortic segment divided by the aortic pulse pressure (*ΔP*) measured by a sphygmomanometer [96]. Automatic measurement of aortic cross-sectional area is also possible [97].

$$\begin{array}{c} \text{Aortic}\;\text{distensibility}=\left(\text{Amax-Amin}\right)/\text{Amin}\\ \;\times \text{pulse}\;\text{pressure},\\ \begin{array}{c} \;\text{Amax}=\text{maximal}\;\text{aortic}\;\text{area}\;{\mathrm{mm}}^{2},\\ \;\text{Amin}=\text{minimum}\;\text{aortic}\;\text{area}\;{\mathrm{mm}}^{2}, \end{array}\\ \;\text{Pulse}\;\text{pressure}=\text{systolic}-\text{diastolic}\;\text{blood}\;\text{pressure}\\ \;\left(\text{mmHg}\right). \end{array}$$

The accuracy of the indirect measurement of the pressure change needed to compute distensibility is limited as it ignores the changes in the pressure wave as it propagates through the arterial tree (a process known as amplification). Further, it is important to obtain this pressure data on patients ideally lying in the CMR scanner using CMR compatible apparatus. Despite the limitations of the pressure measurement, there is a good correlation between measurement of local aortic compliance and measurement of global compliance from the speed of the propagation of the flow wave within the vessel [98].

Flow wave velocity is defined as the speed with which a flow wave propagates along a vessel and is regarded as the purest measure of arterial stiffness. It is the quotient of distance travelled divided by the time taken for the flow wave to move between the two points and represents an average for that length of vessel. The approach is dependent on assessment of path-length travelled and accurate measurement of pulse arrival time. The latter requires recognition of equivalent features or points on leading edges of the proximal and distal flow waveforms, a process made complicated by alterations that occur in flow wave morphology and magnitude as it progresses down the vessel. Unlike non-invasive measurements relying on linear, transcutaneous measurements, CMR makes no assumptions about the shape of the artery and can accurately measure the path-length travelled.

### Follow-up studies

What is most important in assessment of the aorta is accuracy and reproducibility. These patients are attending for lifelong follow-up and variation in measurement techniques could have disastrous consequences.

In CMR there is a surprising lack of standardisation of methodologies. In part this stems from the flexibility of the technique, with multiple types of sequences offering the ability to derive information about the aorta. For example, ECG-gated end-diastolic black-blood images using spin echo can be used to assess anatomy and morphology; SSFP-based cine images can be used to do the same. Contrast-enhanced MRA may be used but this is usually done without ECG-gating and there is a risk of motion artefact, particularly in the aortic root.

With such a wide choice available one should be aware of the advantages, limitations and differences between the sequences and the measuring planes. In recent years a body of work has been undertaken to investigate this further.

**Figure 14:**
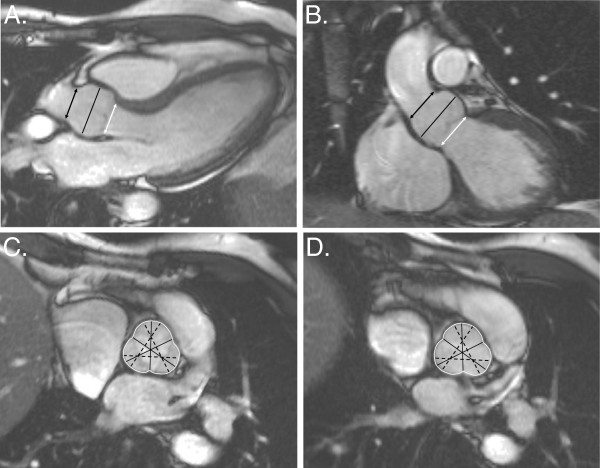
**SSFP-based imaging. A**, An oblique sagittal LVOT cine at end diastole showing the levels of annulus, sinus, and sinotubular junction measurements (white arrow, black line, and black arrow, respectively). **B**, An oblique coronal LVOT cine showing the equivalent levels of measurement. **C**, A systolic sinus plane image, showing 3 cusp-commissure and 3 cusp-cusp lines of measurement (continuous and dashed black lines, respectively) with the cross-sectional area outlined in white. **D**, An end diastolic sinus plane image, showing the cusp-commissure and cusp-cusp lines of measurement. Reproduced with permission [99].

Measurements of the aortic root can reliably be obtained from SSFP-based cine images. Sinus of valsalva planes can be planned from oblique sagittal and oblique coronal LVOT planes. Sinus-sinus and sinus-commissure measurements taken at end-diastole are most comparable with echo and reference values are available (Figure 14) [99], It is important to quote the maximum trans-sinuses measurement which is usually the sinus-sinus measurement in the report.

It is well recognised that aortic size can be significantly overestimated by axial measurements when compared with orthogonal measurements when the aorta is not straight and the axial plane is not perpendicular to the true axis of the aorta. This could affect management decisions in up to 13% patients and has led some investigators to advocate the use of contrast-enhanced magnetic resonance angiography (CE-MRA) in patients, where possible with ECG-gating to minimise aortic root motion-induced artefacts. However, this appears to be rarely done in clinical practice [100]. When CE-MRA is used, careful reconstruction of images is required. Unfortunately without ECG-gating it is the root measurements which are the least reliable, but normal values have been generated for various patient groups [101].

In response to concerns about regularly performing CE-MRA with gadolinium in patients, a non contrast-enhanced MRA using 3D-navigated SSFP has been trialled [102]. Although it has had some success, it is susceptible to arrhythmia and adds approximately 10 minutes to the scan time. Comparison of 3D-navigated SSFP with CE-MRA, 2D T2 black blood (BB) and 2D SSFP-based cines has been reported. [103] 3D-navigated SSFP and CE-MRA provide the largest aortic measurements and T2 BB the smallest. T2 BB has the best inter-observer variability but all sequences have an inter-observer variability of >0.9. Vessel wall analysis is optimal on T2 BB images. 3D-navigated SSFP measurements correlated best with the gold standard of ECG-gated CT, but the images are less sensitive for demonstrating dissection flaps when compared with CE-MRA.

It is reasonable to derive from these studies that several sequences should be used to assess the aorta in patients with MFS. Our ‘basic’ T2 BB and 2D SSFP sequences can produce beautiful images from which extremely detailed and reliable measurements can be made [104]. However CE-MRA can be of particular use when assessing a tortuous aorta and navigated 3D-SSFP may be used in those in whom renal impairment is an issue.

### Clinical application

The following sections contain examples of black blood, SSFP and CE-MRA images used in clinical context.

### Aorta and branches

**Figure 15:**
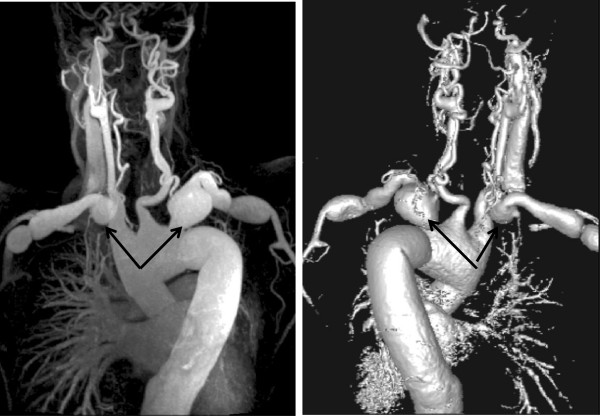
CE-MRA Demonstrating Bilateral Subclavian Artery Aneurysms in a Patient who has undergone Previous Aortic Root Surgery, examples highlighted by arrows.

**Figure 16:**
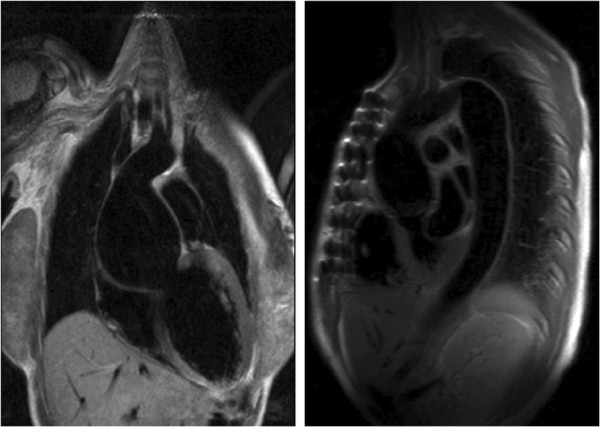
**Black blood (spin echo) imaging before (coronal) and after (sagittal) emergency VSARR for type A dissection. **Note the significant reduction in aortic root dimensions.

**Figure 17:**
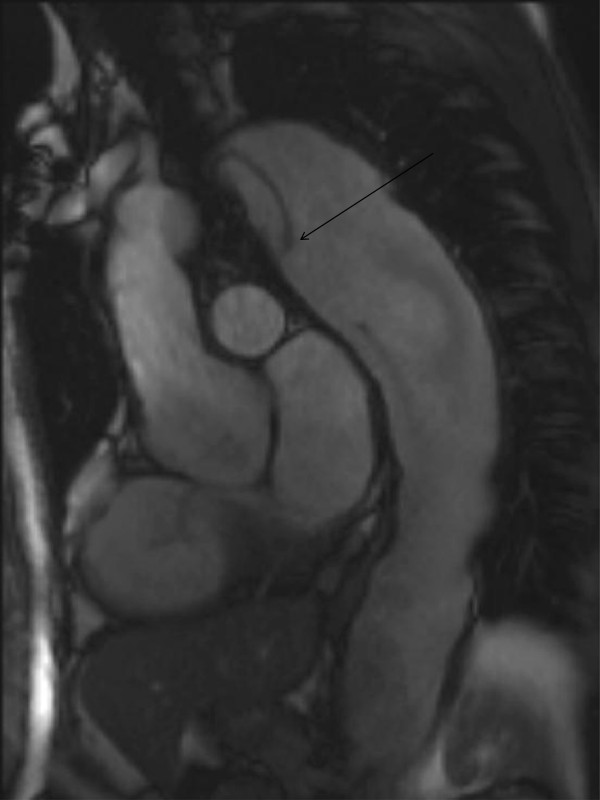
**SSFP sagittal oblique image following emergency repair of type A dissection- residual dissection in proximal arch extending into right common carotid artery.** Communication between true and false lumens in proximal descending aorta is evident. Dissection flap highlighted by arrow.

A description of the classification of aortic dissection and complications is given in the section on the aorta. Figure 15 demonstrates aneurysmal involvement of the subclavian arteries in a patient who has undergone previous surgery. Figure 16 demonstrates black blood imaging of a type A dissection and Figure 17 demonstrates post-operative SSFP imaging of a dissection which involved the ascending aorta, but also the head and neck vessels with a visible flap in the proximal descending aorta.

### Valves

Whilst the primary purpose of CMR in MFS is usually aortic assessment, it is also capable of providing useful information on valvular function. SSFP-based cine imaging in an LVOT view can reliably detect mitral valve prolapse [105]. The anatomical basis for regurgitation can then be investigated by performing a mitral valve stack. This is a contiguous stack of 5 mm slices aligned with MV inflow and cutting through the major line of coaptation. It should move from the superior to inferior commissure in the LVOT view. In this way the scallops of both leaflets may be demonstrated. Any regurgitation may be quantified:

$$\begin{array}{l} \text{Mitral}\;\text{regurgitant}\;\text{volume}\\ \;=\mathrm{LV}\;\text{stroke}\;\text{volume}-\text{aortic}\;\text{forward}\;\text{flow}\\ \;\left(\mathrm{ml}/\text{cardiac}\;\text{cycle}\;\text{from}\text{aortic}\;\text{flow}\;\text{analysis}\;\mathrm{at}\right)\\ \;\left(\text{level}\;\mathrm{of}\;\text{the}\;\text{STJ}\right) \end{array}$$

$$\begin{array}{l} \text{Regurgitant}\;\text{fraction}\\ \;=\text{regurgitant}\;\text{volume}/\mathrm{LV}\;\text{stroke}\;\text{volume}\times 100\% \end{array}$$

The aortic valve should be assessed well by a standard CMR protocol. The LVOT and coronal LVOT SSFP-based cines will demonstrate AR as a signal void. The ‘en face’ AV view planned from the LVOT views will demonstrate abnormal cusp morphology or defects in coaptation. However cine images alone should never be used to quantify any degree of regurgitation.

Through-plane velocity flow mapping at the STJ should be performed to quantitatively assess AR.

Aortic regurgitant volume in ml/cardiac cycle is the reverse flow from analysis of flow mapping.

$$\begin{array}{l} \text{Regurgitant}\;\text{fraction}\\ \;=\text{regurgitant}\;\text{volume}/\text{aortic}\;\text{forward}\;\text{flow}\;\mathrm{in}\\ \;\text{systole}\times 100\% \end{array}$$

**Figure 18:**
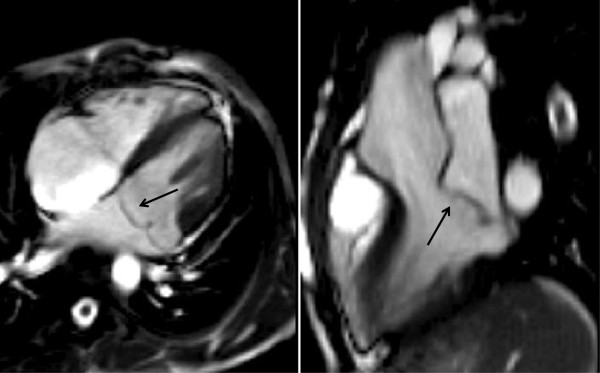
Bileaflet prolapse of the mitral valve at end systole (see arrow) on 4-chamber and LVOT SSFP images.

**Figure 19:**
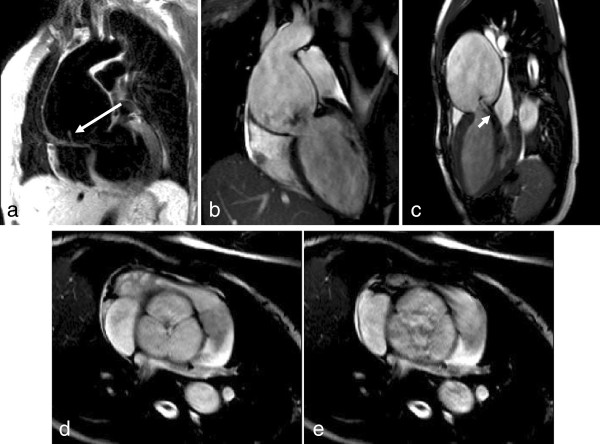
T1 weighted spin echo image (a) and SSFP imaging in diastole (b-d) and systole (e) of classical aortic root dilatation with type A dissection (large arrow) and functional severe eccentric AR (short arrow) before repair.

**Figure 20:**
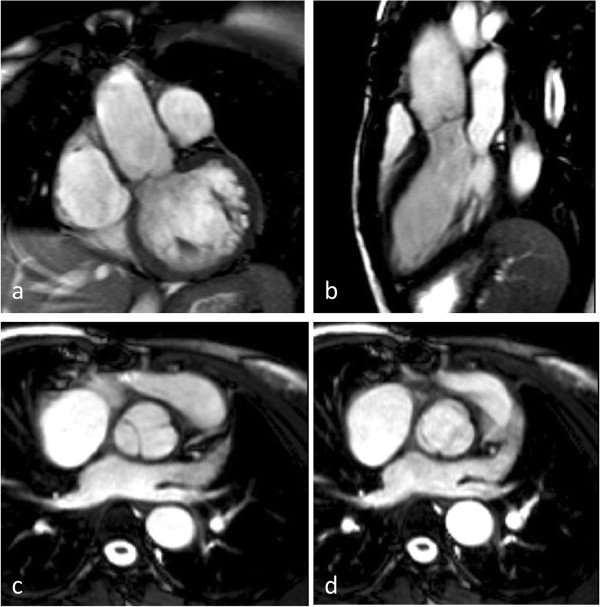
Diastolic (a-c) and systolic (d) frames from complete cine SSFP imaging showing appearance after successful valve- sparing surgery.

This method will underestimate the degree of AR since the slice is at the level of the ST-J and does not account for annular motion and coronary perfusion during the cardiac cycle (see Figures 18,19 and 20).

### Cardiomyopathy/Assessment of ventricular function and volumes

**Figure 21:**
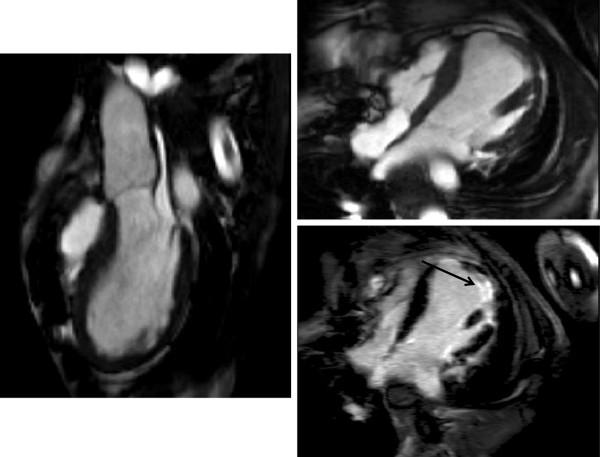
**Anterolateral infarction with transmural enhancement in a patient with a previous aortic root repair. **(SSFP images without contrast left and top; lower image is equivalent 4-chamber image following administration of gadolinium. Arrow demonstrates transmural enhancement).

CMR is the gold standard for biventricular assessment and can detect the mild ‘DCM phenotype’ of MFS cardiomyopathy (see myocardial involvement in MFS). Several CMR software packages allow rapid analysis of biventricular volumes and function. If a more focused approach is required clinically then LV analysis can be performed. If LVEF is abnormal, in the absence of valvular dysfunction or other aetiology, then RV analysis should be performed [53]. Notably, ventricular impairment due to MFS is usually global, hence any regional dysfunction warrants further clinical investigation and tissue characterisation eg with LGE to exclude concomitant pathology, (see Figure 21).

### Post surgical assessment

After intervention or open surgery, the American guidelines advocate the use of CT to detect asymptomatic leaks or pseudoaneurysms if metallic surgical devices are likely to cause significant artefact. On a pre-discharge CT, non-contrast images may be used to identify surgical materials which can be difficult to distinguish from leaks on contrast-enhanced images. However use of retrospective gating means this comes with an increased dose of radiation [91].

When artefact is not an issue, and usually it is not, then CMR may be used. CMR has been used very successfully to identify complications after prosthetic replacement of the ascending aorta for over a decade [106]. A post–operative study within the index admission is advised, followed by repeats at 1,3,6 and 12 months and annually thereafter [33]. The standard protocol suggested earlier would allow comprehensive post-operative assessment.

Peri-prosthetic leaks and haemopericardium can be demonstrated on all static images and the sources determined on subsequent SSFP and velocity encoded phase imaging. Using SSFP-based cine imaging aortic measurements may be made and any residual dissection flap visualised. Slow flow and thrombus can be identified and their presence confirmed on TSE sequences [107]. If necessary, through-plane flow mapping can be used to assess true luminal flow. CMR has the added value of easily quantifying post-operative biventricular function unhindered by chest-wall anatomy. If renal function permits, then LGE should be performed (see below).

It is essential to corroborate findings with the operative notes and TOE and postoperative TTE. By doing so any graft angles or tied-off cannulation sites should not be mistaken for further dissection or pseudoaneurysm formation. In addition, proximal and distal graft anastomoses and coronary reimplantation sites can be identified.

**Figure 22:**
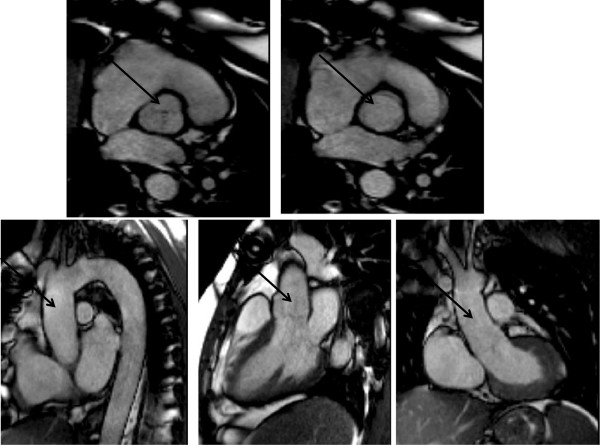
**Normal appearance of a VSARR on SSFP-based imaging. **The upper panels demonstrate the aortic valve in diastole and systole; lower panels demonstrate aortic appearance.

**Figure 23:**
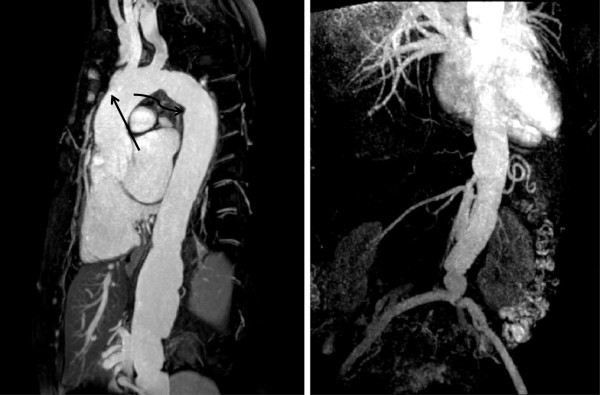
**Tortuous abdominal aorta, previous VSARR and thoracoabdominal graft. **Root replacement anastomosed to upper ascending aorta proximal to brachiocephalic artery (straight arrow). Proximal descending arch is anastomosed to thoracoabdominal gaft (curved arrow) on CE-MRA.

**Figure 24:**
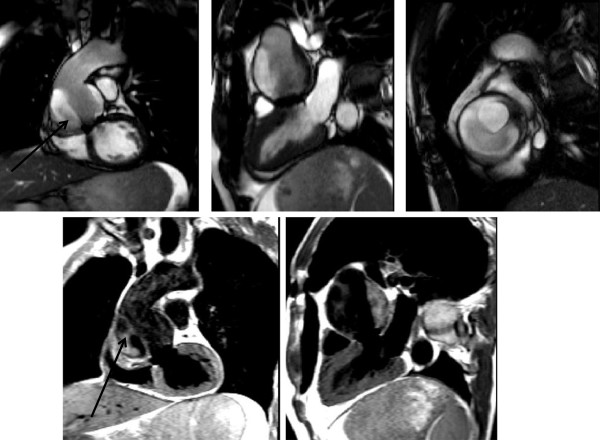
Haematoma outwith graft following a Bentall procedure on SSFP (coronal, sagittal and transaxial in upper panel) and black blood imaging (lower panel).

**Figure 25:**
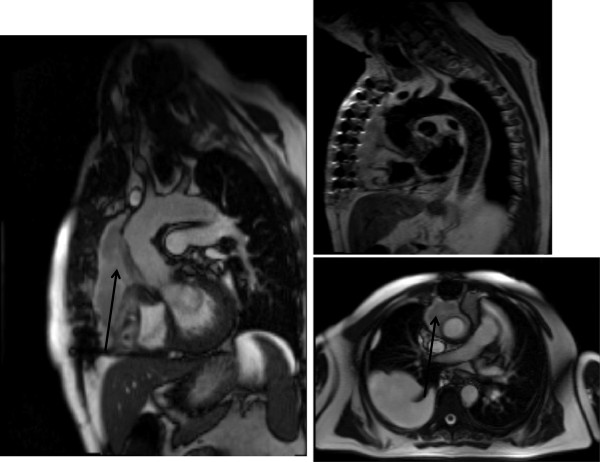
Para-aortic haematoma (arrowed) – previous aortic root homograft and re-do surgery (SSFP imaging in sagittal view on the left, sagittal TSE BB in upper right, SSFP imaging lower right in transaxial cut, also demonstrating right pleural effusion).

**Figure 26:**
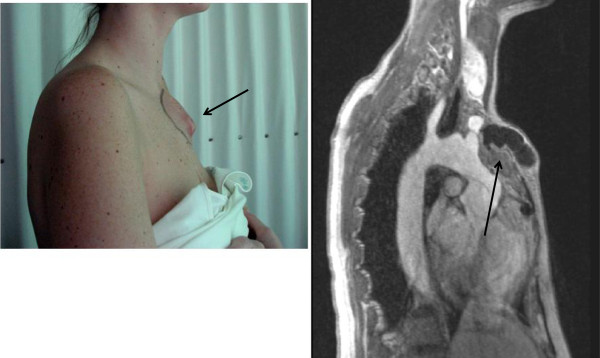
Subcutaneous infected collection visible externally and on CMR, courtesy of Dr Sonya Babu-Narayan.

Following a VSARR, imaging serves a number of important roles:

1. Define post-operative anatomy including surgical materials e.g. graft, Teflon strips, pledgets, Bioglue.

2. Identify haematoma or leak at proximal and distal graft anastomoses and coronary reimplantation sites. This may be localised or take the form of extensive haemopericardium.

3. Assess valvular function and look for paravalvular leaks.

4. Assess ventricular function. Perioperative myocardial infarction secondary to prolonged hypotension or difficulties with coronary reimplantation has been reported and should be readily defined on LGE.

5. Look for residual dissection and thrombus - a patent or partially thrombosed false lumen at the time of primary surgical repair is known to increase this risk [108].

6. Look for infection – either graft or sternum (See Figures 22, 23, 24, 25 and 26)

### Additional notes on assessment of the biophysical properties of the aortic wall and the future

Without question CMR is an elegant technique for combining anatomical and functional assessment. In MFS this can be exploited further to assess the biophysical properties of the aortic wall [109].

The aorta is more than just a conduit for passive blood flow. It is an elastic tube, the diameter of which varies in response to haemodynamic forces, transforming the pulsatile ventricular output into a continuous flow of blood to the peripheries. This ‘distensibility’ depends in large part on the elastic content, arrangement and maintenance of the vessel wall which is altered in MFS.

CMR is well placed for the assessment of aortic distensibility, the method is described and illustrated in the CMR protocol section. This technique has been validated against echocardiographic assessments in several studies [110,111]. However, CMR has superior anatomical reproducibility in terms of anatomical landmarks and hence is more valuable and reproducible in terms of follow up. The process by which aortic contouring is performed is still being refined [112].

Compliance is the reciprocal of the resistance to deformation and is defined as the change in volume per unit change in pressure (microml/mm Hg). Regional aortic compliance has been studied by CMR, using a formula similar to that for assessment of distensibility, but using slice thickness to calculate the volume of blood in each aortic segment studied [96].

**Figure 27:**
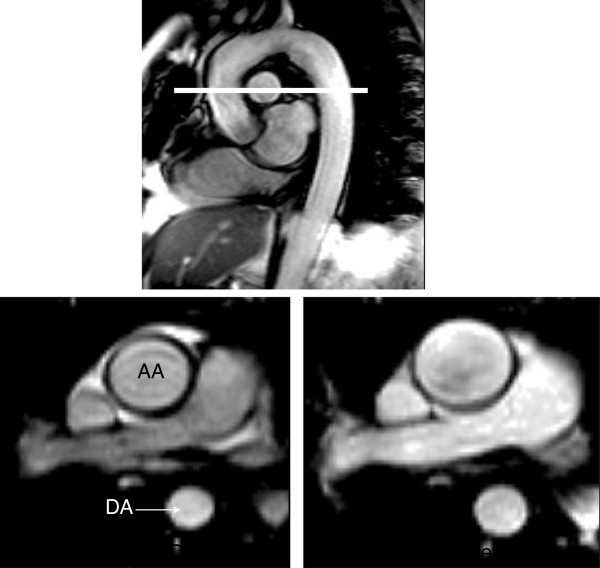
**Upper panel shows an oblique sagittal image of the aorta demonstrating sites where flow wave velocity and regional compliance are measured. **The lower images represent the oblique transverse plane in diastole (left) and systole (right), in a healthy individual. (AA ascending aorta; DA descending aorta).

**Figure 28:**
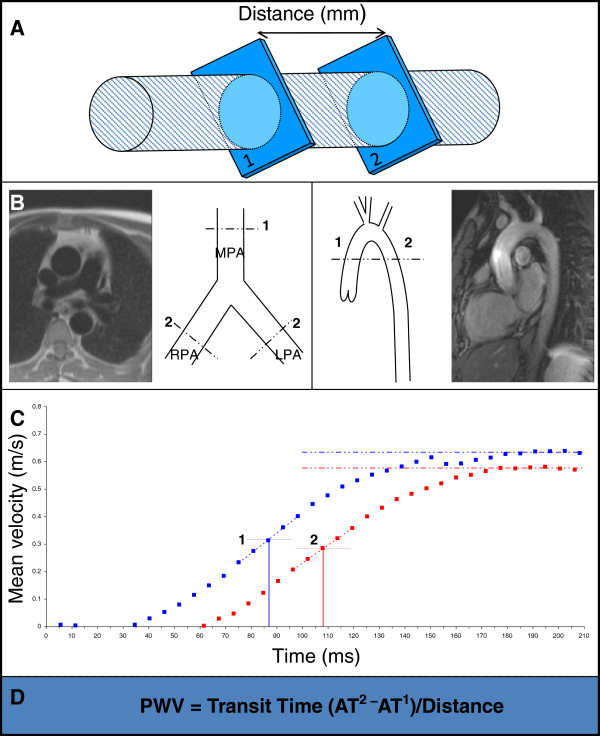
**Demonstrates assessment of pulse wave velocity. **Phase velocity acquisition across a vessel is undertaken at points 1 and 2 and the distance between them is measured. Slice prescription for the proximal pulmonary arteries and the aorta are both demonstrated. The descending and ascending aortic locations are obtained within a single slice. Transit time is defined by the difference in arrival time of the flow wave at points 1 and 2 and divided by the distance to give pulse wave velocity. (MPA main pulmonary artery; LPA left pulmonary artery; RPA right pulmonary artery). Reproduced with permission from Cardiovascular Magnetic Resonance, Second edition, Manning WJ and Pennell DJ.

At a systemic arterial level compliance has been assessed echocardiographically in patients with MFS and used to assess response to pharmacotherapy [22,85]. In this context compliance is not assessed by means of pressure changes, but rather by the speed of propagation of the pulse wave velocity (PWV), which is generally higher as distensibility decreases. This speed of propagation is regarded as the purest measure of arterial stiffness. In the aorta it is usually calculated as the ratio between the distance separating 2 points of interest and the time taken for a pressure/velocity wave to travel this distance. The feasibility of this measurement by CMR was demonstrated over 15 years ago [98]. With its unlimited views, CMR is well placed to accurately assess the length of aorta travelled by the PWV, even when chest wall or aortic anatomy is distorted. Exploiting the aortic anatomy it is possible to select an anatomic slice, usually at the level of the pulmonary bifurcation, from which a phase contrast/velocity encoded image can be acquired, in a plane perpendicular to the aortic lumen. In this one slice, phase velocity maps which can be used for quantifying blood volume flowing through the imaging plane, can be generated for 2 aortic levels [113] (See Figures 27 and 28).

Although multiple flow imaging techniques have been tried in CMR, it is the phase velocity/velocity encoding technique which is the most utilised. However, the optimal method for estimation of transit time from the resultant velocity curves remains under review [114]. Research is currently focused on a revision of the traditional 2-slice method to a two-directional in-plane velocity-encoded CMR covering the entire aorta in 3 parallel oblique-sagittal slices and, more recently, to a 4-slice breath-hold through-plane velocity-encoded CMR [115,116]. This technique may be particularly useful in predicting lack of luminal growth in the ascending aorta [117].

Indeed for some time investigators have recognised the importance of both aortic diameter and distensibility in predicting aortic events in those with MFS [118]. For the aortic root and abdominal aorta it is the initial diameter which is the major predictor of progressive dilatation and dissection, although distensibility is also reduced; for the thoracic descending aorta, local distensibility is an independent predictor. In one study a cut off of a distensibility of 3.1 × 10^-3^ mmHg ^-1^ was found to have sensitivity of 100% and specificity of 56% for lack of progressive dilatation. An annual assessment of aortic distensibility as well as diameters at 4 aortic levels has been proposed [119]. In addition it is possible to assess changes in aortic distensibility and PWV in response to beta-blockade in patients with MFS, although such an approach has not become routine clinical practice [120]. (It is worth noting that in those with MFS, even though beta-blockade significantly increases aortic root distensibility, it still remains significantly less than in control subjects in this study.) Other studies have demonstrated a heterogeneous response to beta-blockade with regard to aortic elasticity and, in these individuals, a combination of CMR assessment of aortic biophysical properties and blood pressure measurements may demonstrate that the aorta is already ‘under strain’, utilising not only elastin but also collagen to support daily ‘load bearing’. These patients would be less likely to benefit from beta-blockade [121].

Distensibility may even be used as a screening tool in family members who do not appear to have the MFS phenotype [122]. Similarly, parameters of aortic compliance can be abnormal in young patients with MFS and normal aortic dimensions [123]. Although in those with MFS, distensibility may be abnormal in most parts of the aorta which are studied, it is possible that the values obtained in some segments may overlap with the normal range. In these situations, the assessment of the PWV will demonstrate the abnormalities [109]. However, a comprehensive assessment of distensibility at several aortic levels, including the aortic root, is also likely to demonstrate abnormalities in an individual with MFS and hence in a pragmatic clinical study this is an appropriate and reproducible undertaking. Of note, aging has a progressive negative effect on aortic compliance. However, in MFS, the values obtained are significantly different from healthy controls of an equivalent age [124].

CMR is uniquely placed to assess morphology whilst obtaining ecg-gated and spatially-encoded functional information. Therefore attention has now turned to more detailed analysis of aortic flow in MFS by means of 4D (time-resolved 3D) phase-contrast CMR [125-128]. How this contributes to negative aortic modelling has yet to be elucidated but is the subject of ongoing work [129]. In patients with MFS this technique has been used to demonstrate aortic flow disturbance and to propose a new flow-based classification of aortic disease in those with MFS [130]. Previous alternatives to this approach involved using CMR-derived parameters of the aortic wall combined with computational fluid dynamics [131] or CMR-derived aortic anatomical images reconstructed and subjected to finite element analysis to determine wall stress and hence risk of rupture [132]. The future may be one of combining the assessment of an individual’s biophysical wall properties with an assessment of individual flow patterns and shear stress to refine the timing of surgical intervention and the success of both pharmacological and surgical intervention [72,125]. However this technique remains a research tool at present whilst its applications and limitations are fully elucidated [128,133].

## Conclusion

All guidelines recommend lifelong follow-up of the entire aorta in patients with MFS. Yet data from the Euro Heart Survey on adult congenital heart disease demonstrates that within Europe we are falling well short of this [40]. Our goal is to best utilise the multimodality techniques at our disposal to be accurate, reproducible and accessible to those with MFS. CMR is an integral part of the imaging in MFS, which dictates the timing and nature of intervention and hence dictates our patients’ outcomes.

## Consent

Written informed consent was obtained from the patient for the photographic image used in this review.

## Competing interest

The authors have no conflict of interest to declare.

## Authors’ contributions

HD drafted the manuscript and contributed to the figures, RHM provided figures and contributed to the manuscript and its revision. All authors read and approved the final manuscript.

## References

[B1] Ho NC Tran JR Bektas A Marfan’s syndrome Lancet 2005 366 9501 1978 1981 10.1016/S0140-6736(05)66995-4 16325702

[B2] Beighton P de Paepe A Danks D Finidori G Gedde-Dahl T Goodman R International nosology of heritable disorders of connective tissue, Berlin, 1986 Am J Med Genet 1988 29 3 581 594 10.1002/ajmg.1320290316 3287925

[B3] De Paepe A Devereux RB Dietz HC Hennekam RC Pyeritz RE Revised diagnostic criteria for the Marfan syndrome Am J Med Genet 1996 62 4 417 426 10.1002/(SICI)1096-8628(19960424)62:4<417::AID-AJMG15>3.0.CO;2-R 8723076

[B4] Loeys BL Dietz HC Braverman AC Callewaert BL De Backer J Devereux RB The revised Ghent nosology for the Marfan syndrome J Med Genet 2010 47 7 476 485 10.1136/jmg.2009.072785 20591885

[B5] Pyeritz RE McKusick VA The Marfan syndrome: diagnosis and management N Engl J Med 1979 300 14 772 777 10.1056/NEJM197904053001406 370588

[B6] Keane MG Pyeritz RE Medical management of Marfan syndrome Circulation 2008 117 21 2802 2813 10.1161/CIRCULATIONAHA.107.693523 18506019

[B7] Hilhorst-Hofstee Y Rijlaarsdam ME Scholte AJ Swart-van den Berg M Versteegh MI van der Schoot-van Velzen I The clinical spectrum of missense mutations of the first aspartic acid of cbEGF-like domains in fibrillin-1 including a recessive family Hum Mutat 2010 31 12 E1915 E1927 10.1002/humu.21372 20886638 PMC3051827

[B8] Kainulainen K Pulkkinen L Savolainen A Kaitila I Peltonen L Location on chromosome 15 of the gene defect causing Marfan syndrome N Engl J Med 1990 323 14 935 939 10.1056/NEJM199010043231402 2402262

[B9] Robinson PN Godfrey M The molecular genetics of Marfan syndrome and related microfibrillopathies J Med Genet 2000 37 1 9 25 10.1136/jmg.37.1.9 10633129 PMC1734449

[B10] Faivre L Collod-Beroud G Child A Callewaert B Loeys BL Binquet C Contribution of molecular analyses in diagnosing Marfan syndrome and type I fibrillinopathies: an international study of 1009 probands J Med Genet 2008 45 6 384 390 10.1136/jmg.2007.056382 18310266

[B11] Mc KV The cardiovascular aspects of Marfan’s syndrome: a heritable disorder of connective tissue Circulation 1955 11 3 321 342 10.1161/01.CIR.11.3.321 14352380

[B12] Jondeau G Michel JB Boileau C The translational science of Marfan syndrome Heart 2011 97 15 1206 1214 10.1136/hrt.2010.212100 21742617

[B13] Habashi JP Judge DP Holm TM Cohn RD Loeys BL Cooper TK Losartan, an AT1 antagonist, prevents aortic aneurysm in a mouse model of Marfan syndrome Science 2006 312 5770 117 121 10.1126/science.1124287 16601194 PMC1482474

[B14] Ng CM Cheng A Myers LA Martinez-Murillo F Jie C Bedja D TGF-beta-dependent pathogenesis of mitral valve prolapse in a mouse model of Marfan syndrome J Clin Invest 2004 114 11 1586 1592 15546004 10.1172/JCI22715 PMC529498

[B15] Loeys BL Schwarze U Holm T Callewaert BL Thomas GH Pannu H Aneurysm syndromes caused by mutations in the TGF-beta receptor N Engl J Med 2006 355 8 788 798 10.1056/NEJMoa055695 16928994

[B16] Albornoz G Coady MA Roberts M Davies RR Tranquilli M Rizzo JA Familial thoracic aortic aneurysms and dissections–incidence, modes of inheritance, and phenotypic patterns Ann Thorac Surg 2006 82 4 1400 1405 10.1016/j.athoracsur.2006.04.098 16996941

[B17] Simpson CF Kling JM Palmer RF Beta-aminopropionitrile-induced dissecting aneurysms of turkeys: treatment with propranolol Toxicol Appl Pharmacol 1970 16 1 143 153 10.1016/0041-008X(70)90170-5 5416744

[B18] Ose L McKusick VA Prophylactiv use of propranolol in the Marfan syndrome to prevent aortic dissection Birth Defects Orig Artic Ser 1977 13 3C 163 169 19114

[B19] Shores J Berger KR Murphy EA Pyeritz RE Progression of aortic dilatation and the benefit of long-term beta-adrenergic blockade in Marfan’s syndrome N Engl J Med 1994 330 19 1335 1341 10.1056/NEJM199405123301902 8152445

[B20] Danyi P Elefteriades JA Jovin IS Medical therapy of thoracic aortic aneurysms: are we there yet? Circulation 2011 124 13 1469 1476 10.1161/CIRCULATIONAHA.110.006486 21947934

[B21] Brooke BS Habashi JP Judge DP Patel N Loeys B Dietz HC 3rd Angiotensin II blockade and aortic-root dilation in Marfan’s syndrome N Engl J Med 2008 358 26 2787 2795 10.1056/NEJMoa0706585 18579813 PMC2692965

[B22] Ahimastos AA Aggarwal A D’Orsa KM Formosa MF White AJ Savarirayan R Effect of perindopril on large artery stiffness and aortic root diameter in patients with Marfan syndrome: a randomized controlled trial JAMA 2007 298 13 1539 1547 10.1001/jama.298.13.1539 17911499

[B23] Maron BJ Chaitman BR Ackerman A Bayes de Luna MJ Corrado D Crosson JE Recommendations for physical activity and recreational sports participation for young patients with genetic cardiovascular diseases Circulation 2004 109 22 2807 2816 10.1161/01.CIR.0000128363.85581.E1 15184297

[B24] Murdoch JL Walker BA Halpern BL Kuzma JW McKusick VA Life expectancy and causes of death in the Marfan syndrome N Engl J Med 1972 286 15 804 808 10.1056/NEJM197204132861502 5011789

[B25] Krause KJ Marfan syndrome: literature review of mortality studies J Insur Med 2000 32 2 79 88 15912906

[B26] Silverman DI Burton KJ Gray J Bosner MS Kouchoukos NT Roman MJ Boxer M Devereux RB Tsipouras P Life expectancy in the marfan syndrome Am J Cardiol 1995 75 157 160 10.1016/S0002-9149(00)80066-1 7810492

[B27] Silverman DI Gray J Roman MJ Bridges A Burton K Boxer M Family history of severe cardiovascular disease in Marfan syndrome is associated with increased aortic diameter and decreased survival J Am Coll Cardiol 1995 26 4 1062 1067 10.1016/0735-1097(95)00258-0 7560600

[B28] Etter LEGLP Arachnodactyly complicated by dislocated lens and death from rupture of dissecting aneurysm of the aorta JAMA 1943 123 88 89 10.1001/jama.1943.82840370001006

[B29] Zagrosek AVK-BF Polleichtner S Schaarschmidt Schulz-Menger J Hemodynamic impact of surgical correction of pectus excavatum - a cardiovascular magnetic resonance study J Cardiovasc Magn Reson 2011 13 Suppl 1 190 10.1186/1532-429X-13-S1-P190

[B30] Saleh RS Finn JP Fenchel M Moghadam AN Krishnam M Abrazado M Cardiovascular magnetic resonance in patients with pectus excavatum compared with normal controls J Cardiovasc Magn Reson 2010 12 73 10.1186/1532-429X-12-73 21144053 PMC3022801

[B31] Alpendurada F Mohiaddin R 1039 Prevalence of cardiovascular manifestations in patients with Marfan syndrome: a cardiovascular magnetic resonance study J Cardiovasc Magn Reson 2008 10 Suppl 1 A164 10.1186/1532-429X-10-S1-A164

[B32] Detaint D Faivre L Collod-Beroud G Child AH Loeys BL Binquet C Cardiovascular manifestations in men and women carrying a FBN1 mutation Eur Heart J 2010 31 18 2223 2229 10.1093/eurheartj/ehq258 20709720

[B33] Hiratzka LF Bakris GL Beckman JA Bersin RM Carr VF Casey DE Jr 2010 ACCF/AHA/AATS/ACR/ASA/SCA/SCAI/SIR/STS/SVM guidelines for the diagnosis and management of patients with thoracic aortic disease: a report of the American college of cardiology foundation/American heart association task force on practice guidelines, American association for thoracic surgery, American college of radiology, American stroke association, society of cardiovascular anesthesiologists, society for cardiovascular angiography and interventions, society of interventional radiology, society of thoracic surgeons, and society for vascular medicine Circulation 2010 121 13 e266 e369 20233780 10.1161/CIR.0b013e3181d4739e

[B34] Park SW Hutchison S Mehta RH Isselbacher EM Cooper JV Fang J Association of painless acute aortic dissection with increased mortality Mayo Clinic proceedings Mayo Clinic 2004 79 10 1252 1257 10.4065/79.10.1252 15473405

[B35] Khan IA Nair CK Clinical, diagnostic, and management perspectives of aortic dissection Chest 2002 122 1 311 328 10.1378/chest.122.1.311 12114376

[B36] Bossone E Rampoldi V Nienaber CA Trimarchi S Ballotta A Cooper JV Usefulness of pulse deficit to predict in-hospital complications and mortality in patients with acute type A aortic dissection Am J Cardiol 2002 89 7 851 855 10.1016/S0002-9149(02)02198-7 11909573

[B37] Yagdi T Atay Y Engin C Mahmudov R Tetik O Iyem H Impact of organ malperfusion on mortality and morbidity in acute type A aortic dissections J Card Surg 2006 21 4 363 369 10.1111/j.1540-8191.2006.00246.x 16846414

[B38] Finkbohner R Johnston D Crawford ES Coselli J Milewicz DM Marfan syndrome. Long-term survival and complications after aortic aneurysm repair Circulation 1995 91 3 728 733 10.1161/01.CIR.91.3.728 7828300

[B39] Mimoun L Detaint D Hamroun D Arnoult F Delorme G Gautier M Dissection in Marfan syndrome: the importance of the descending aorta Eur Heart J 2011 32 4 443 449 10.1093/eurheartj/ehq434 21147864

[B40] Engelfriet PM Boersma E Tijssen JG Bouma BJ Mulder BJ Beyond the root: dilatation of the distal aorta in Marfan’s syndrome Heart 2006 92 9 1238 1243 10.1136/hrt.2005.081638 16488927 PMC1861183

[B41] Waller BF Clary JD Rohr T Nonneoplastic diseases of aorta and pulmonary trunk–Part V Clin Cardiol 1997 20 12 1026 1028 10.1002/clc.4960201210 9422842 PMC6655277

[B42] De Backer J Loeys B Devos D Dietz H De Sutter J De Paepe A A critical analysis of minor cardiovascular criteria in the diagnostic evaluation of patients with Marfan syndrome Genet Med 2006 8 7 401 408 10.1097/01.gim.0000223550.41849.e3 16845272

[B43] Stuart AG Williams A Marfan’s Syndrome and the heart Arch Dis Child 2007 92 4 351 356 10.1136/adc.2006.097469 17376944 PMC2083669

[B44] Bhudia SK Troughton R Lam BK Rajeswaran J Mills WR Gillinov AM Mitral valve surgery in the adult Marfan syndrome patient Ann Thorac Surg 2006 81 3 843 848 10.1016/j.athoracsur.2005.08.055 16488682

[B45] Gu XHY Li Z Han J Chen J Nixon JV Marfan syndrome: echocardiographic valvular characteristics compared to histological findings. JACC 2011 57 E1410 10.1016/S0735-1097(11)61410-1

[B46] Underwood MJ El Khoury G Deronck D Glineur D Dion R The aortic root: structure, function, and surgical reconstruction Heart 2000 83 4 376 380 10.1136/heart.83.4.376 10722531 PMC1729360

[B47] Fujiseki Y Okuno K Tanaka M Shimada M Takahashi M Kawanishi K Myocardial involvement in the Marfan syndrome Jpn Heart J 1985 26 6 1043 1050 10.1536/ihj.26.1043 3831408

[B48] Kahveci G Erkol A Yilmaz F Dilated cardiomyopathy in a patient with Marfan syndrome accompanied by chronic type A aortic dissection and right atrial thrombus Intern Med 2010 49 23 2583 2586 10.2169/internalmedicine.49.3880 21139296

[B49] Meijboom LJ Timmermans J van Tintelen JP Nollen GJ De Backer J van den Berg MP Evaluation of left ventricular dimensions and function in Marfan’s syndrome without significant valvular regurgitation Am J Cardiol 2005 95 6 795 797 10.1016/j.amjcard.2004.11.042 15757617

[B50] De Backer JF Devos D Segers P Matthys D Francois K Gillebert TC Primary impairment of left ventricular function in Marfan syndrome Int J Cardiol 2006 112 3 353 358 10.1016/j.ijcard.2005.10.010 16316698

[B51] Yetman AT Bornemeier RA McCrindle BW Long-term outcome in patients with Marfan syndrome: is aortic dissection the only cause of sudden death? J Am Coll Cardiol 2003 41 2 329 332 10.1016/S0735-1097(02)02699-2 12535830

[B52] Kiotsekoglou A Sutherland GR Moggridge JC Kapetanakis V Bajpai A Bunce N Impaired right ventricular systolic function demonstrated by reduced atrioventricular plane displacement in adults with Marfan syndrome Eur J Echocardiogr 2009 10 2 295 302 18801726 10.1093/ejechocard/jen239

[B53] Alpendurada F Wong J Kiotsekoglou A Banya W Child A Prasad SK Evidence for Marfan cardiomyopathy Eur J Heart Fail 2010 12 10 1085 1091 10.1093/eurjhf/hfq127 20861133

[B54] Gott VL Greene PS Alejo DE Cameron DE Naftel DC Miller DC Replacement of the aortic root in patients with Marfan’s syndrome N Engl J Med 1999 340 17 1307 1313 10.1056/NEJM199904293401702 10219065

[B55] Coady MA Rizzo JA Hammond GL Mandapati D Darr U Kopf GS What is the appropriate size criterion for resection of thoracic aortic aneurysms? J Thorac Cardiovasc Surg 1997 113 3 476 491 discussion 89–91 10.1016/S0022-5223(97)70360-X 9081092

[B56] Kouchoukos NT Dougenis D Surgery of the thoracic aorta N Engl J Med 1997 336 26 1876 1888 10.1056/NEJM199706263362606 9197217

[B57] Baumgartner H Bonhoeffer P De Groot NM de Haan F Deanfield JE Galie N ESC Guidelines for the management of grown-up congenital heart disease (new version 2010) Eur Heart J 2010 31 23 2915 2957 20801927 10.1093/eurheartj/ehq249

[B58] Davies RR Gallo A Coady MA Tellides G Botta DM Burke B Novel measurement of relative aortic size predicts rupture of thoracic aortic aneurysms Ann Thorac Surg 2006 81 1 169 177 10.1016/j.athoracsur.2005.06.026 16368358

[B59] Meijboom LJ Vos FE Timmermans J Boers GH Zwinderman AH Mulder BJ Pregnancy and aortic root growth in the Marfan syndrome: a prospective study Eur Heart J 2005 26 9 914 920 10.1093/eurheartj/ehi103 15681576

[B60] Bentall H De Bono A A technique for complete replacement of the ascending aorta Thorax 1968 23 4 338 339 10.1136/thx.23.4.338 5664694 PMC471799

[B61] Treasure T The evolution of aortic root surgery for Marfan syndrome Interact Cardiovasc Thorac Surg 2010 10 3 353 355 10.1510/icvts.2010.232611 20118123

[B62] Treasure T Elective replacement of the aortic root in Marfan’s syndrome Br Heart J 1993 69 2 101 103 10.1136/hrt.69.2.101 8435232 PMC1024933

[B63] Choudhary SK Talwar S Kumar AS Bentall operation with valved homograft conduit Tex Heart Inst J 2000 27 4 366 368 11198310 PMC101106

[B64] Bellhouse BJ Bellhouse FH Mechanism of closure of the aortic valve Nature 1968 217 5123 86 87 5635642 10.1038/217086b0

[B65] Dagum P Green GR Nistal FJ Daughters GT Timek TA Foppiano LE Deformational dynamics of the aortic root: modes and physiologic determinants Circulation 1999 100 19 Suppl II54 II62 10567279 10.1161/01.cir.100.suppl_2.ii-54

[B66] Thubrikar M Bosher LP Nolan SP The mechanism of opening of the aortic valve J Thorac Cardiovasc Surg 1979 77 6 863 870 439922

[B67] Kilner PJ Yang GZ Mohiaddin RH Firmin DN Longmore DB Helical and retrograde secondary flow patterns in the aortic arch studied by three-directional magnetic resonance velocity mapping Circulation 1993 88 5 Pt 1 2235 2247 8222118 10.1161/01.cir.88.5.2235

[B68] Yacoub MH Gehle P Chandrasekaran V Birks EJ Child A Radley-Smith R Late results of a valve-preserving operation in patients with aneurysms of the ascending aorta and root J Thorac Cardiovasc Surg 1998 115 5 1080 1090 10.1016/S0022-5223(98)70408-8 9605078

[B69] David TE Armstrong S Ivanov J Feindel CM Omran A Webb G Results of aortic valve-sparing operations J Thorac Cardiovasc Surg 2001 122 1 39 46 10.1067/mtc.2001.112935 11436035

[B70] Bechtel JFMSH Hanke T Charitos EI Schmidtke C Kraatz EG Stierle U Misfeld M Yankah CAWY, Hetzer R Yacoub/David techniques for aortic root opeation: success and failures Aortic root surgery: the biological solution 2009 Germany: Springer

[B71] Miller DC Valve-sparing aortic root replacement: current state of the art and where are we headed? Ann Thorac Surg 2007 83 2 S736 S739 discussion S85-90 10.1016/j.athoracsur.2006.10.101 17257918

[B72] Markl M Draney MT Miller DC Levin JM Williamson EE Pelc NJ Time-resolved three-dimensional magnetic resonance velocity mapping of aortic flow in healthy volunteers and patients after valve-sparing aortic root replacement J Thorac Cardiovasc Surg 2005 130 2 456 463 10.1016/j.jtcvs.2004.08.056 16077413

[B73] Benedetto U Melina G Takkenberg JJ Roscitano A Angeloni E Sinatra R Surgical management of aortic root disease in Marfan syndrome: a systematic review and meta-analysis Heart 2011 97 12 955 958 10.1136/hrt.2010.210286 21228428

[B74] Excellence NIfHaC External Aortic Root Support in Marfan Syndrome Interventional Procedural Guidance 2011 NICE 394 http://publications.nice.org.uk/external-aortic-root-support-in-marfan-syndrome-ipg394/guidance

[B75] Dhillon JS Randhawa GK Straehley CJ McNamara JJ Late rupture after dacron wrapping of aortic aneurysms Circulation 1986 74 3 Pt 2 I11 I14 2943535

[B76] Pepper J John Chan K Gavino J Golesworthy T Mohiaddin R Treasure T External aortic root support for Marfan syndrome: early clinical results in the first 20 recipients with a bespoke implant J R Soc Med 2010 103 9 370 375 10.1258/jrsm.2010.100070 20807993 PMC2930916

[B77] Treasure T Pepper JR Aortic root surgery in Marfan syndrome Heart 2011 97 12 951 952 10.1136/hrt.2010.217240 21357638

[B78] Wang ZG Massimo CG Li M Pan SL Zhang HK Jing W Deployment of endograft in the ascending aorta to reverse type A aortic dissection Asian J Surg 2003 26 2 117 119 10.1016/S1015-9584(09)60232-3 12732497

[B79] Pasic M Hummel M Hetzer R Combined aortic surgery and implantation of a left ventricular assist device N Engl J Med 2002 346 9 711 10.1056/NEJM200202283460918 11870256

[B80] Rajagopal K Rogers JG Lodge AJ Gaca JG McCann RL Milano CA Two-stage total cardioaortic replacement for end-stage heart and aortic disease in Marfan syndrome: case report and review of the literature J Heart Lung Transplant 2009 28 9 958 963 10.1016/j.healun.2009.05.012 19716050

[B81] De Backer J The expanding cardiovascular phenotype of Marfan syndrome Eur J Echocardiogr 2009 10 2 213 215 19106173 10.1093/ejechocard/jen311

[B82] Kramer CM Barkhausen J Flamm SD Kim RJ Nagel E Standardized cardiovascular magnetic resonance imaging (CMR) protocols, society for cardiovascular magnetic resonance: board of trustees task force on standardized protocols J Cardiovasc Magn Reson 2008 10 35 10.1186/1532-429X-10-35 18605997 PMC2467420

[B83] Hundley WG Bluemke D Bogaert JG Friedrich MG Higgins CB Lawson MA Society for cardiovascular magnetic resonance guidelines for reporting cardiovascular magnetic resonance examinations J Cardiovasc Magn Reson 2009 11 5 10.1186/1532-429X-11-5 19257889 PMC2662831

[B84] Kabirdas D Scridon C Brenes JC Hernandez AV Novaro GM Asher CR Accuracy of transthoracic echocardiography for the measurement of the ascending aorta: comparison with transesophageal echocardiography Clin Cardiol 2010 33 8 502 507 10.1002/clc.20807 20734448 PMC6653708

[B85] Kiotsekoglou A Moggridge JC Saha SK Kapetanakis V Govindan M Alpendurada F Assessment of aortic stiffness in marfan syndrome using two-dimensional and Doppler echocardiography Echocardiography 2011 28 1 29 37 10.1111/j.1540-8175.2010.01241.x 21198821

[B86] Kiotsekoglou A Sutherland GR Moggridge JC Nassiri DK Camm AJ Child AH The unravelling of primary myocardial impairment in Marfan syndrome by modern echocardiography Heart 2009 95 19 1561 1566 10.1136/hrt.2008.152934 19224905

[B87] Shiga T Wajima Z Apfel CC Inoue T Ohe Y Diagnostic accuracy of transesophageal echocardiography, helical computed tomography, and magnetic resonance imaging for suspected thoracic aortic dissection: systematic review and meta-analysis Arch Intern Med 2006 166 13 1350 1356 10.1001/archinte.166.13.1350 16831999

[B88] Hagan PG Nienaber CA Isselbacher EM Bruckman D Karavite DJ Russman PL The international registry of acute aortic dissection (IRAD): new insights into an old disease JAMA 2000 283 7 897 903 10.1001/jama.283.7.897 10685714

[B89] LePage MA Quint LE Sonnad SS Deeb GM Williams DM Aortic dissection: CT features that distinguish true lumen from false lumen AJR Am J Roentgenol 2001 177 1 207 211 10.2214/ajr.177.1.1770207 11418429

[B90] Lin FY Devereux RB Roman MJ Meng J Jow VM Jacobs A Assessment of the thoracic aorta by multidetector computed tomography: age- and sex-specific reference values in adults without evident cardiovascular disease J Cardiovasc Comput Tomogr 2008 2 5 298 308 10.1016/j.jcct.2008.08.002 19083966

[B91] Fleischmann D Liang DH Mitchell RS Miller DC Pre- and postoperative imaging of the aortic root for valve-sparing aortic root repair (V-SARR) Semin Thorac Cardiovasc Surg 2008 20 4 365 373 10.1053/j.semtcvs.2008.11.009 19251178

[B92] Feuchtner GM Alkadhi H Karlo C Sarwar A Meier A Dichtl W Cardiac CT angiography for the diagnosis of mitral valve prolapse: comparison with echocardiography1 Radiology 2010 254 2 374 383 10.1148/radiol.2541090393 20093510

[B93] Ha HI Seo JB Lee SH Kang JW Goo HW Lim TH Imaging of Marfan syndrome: multisystemic manifestations Radiographics 2007 27 4 989 1004 10.1148/rg.274065171 17620463

[B94] Farrelly C Davarpanah A Keeling AN Sheehan J Ragin A Yaghmai V Low dose dual-source CT angiography of the thoracic aorta Int J Cardiovasc Imaging 2011 27 7 1025 1034 10.1007/s10554-010-9742-9 21046253

[B95] Meijboom LJ Groenink M van der Wall EE Romkes H Stoker J Mulder BJ Aortic root asymmetry in marfan patients; evaluation by magnetic resonance imaging and comparison with standard echocardiography Int J Card Imaging 2000 16 3 161 168 10.1023/A:1006429603062 11144769

[B96] Mohiaddin RH Underwood SR Bogren HG Firmin DN Klipstein RH Rees RS Regional aortic compliance studied by magnetic resonance imaging: the effects of age, training, and coronary artery disease Br Heart J 1989 62 2 90 96 10.1136/hrt.62.2.90 2765331 PMC1216740

[B97] Rueckert D Burger P Forbat SM Mohiaddin RD Yang GZ Automatic tracking of the aorta in cardiovascular MR images using deformable models IEEE Trans Med Imaging 1997 16 5 581 590 10.1109/42.640747 9368113

[B98] Mohiaddin RH Firmin DN Longmore DB Age-related changes of human aortic flow wave velocity measured noninvasively by magnetic resonance imaging J Appl Physiol 1993 74 1 492 497 8444734 10.1152/jappl.1993.74.1.492

[B99] Burman ED Keegan J Kilner PJ Aortic root measurement by cardiovascular magnetic resonance: specification of planes and lines of measurement and corresponding normal values Circ Cardiovasc Imaging 2008 1 2 104 113 10.1161/CIRCIMAGING.108.768911 19808527

[B100] Bireley WR 2nd Diniz LO Groves EM Dill K Carroll TJ Carr JC Orthogonal measurement of thoracic aorta luminal diameter using ECG-gated high-resolution contrast-enhanced MR angiography J Magn Reson Imaging 2007 26 6 1480 1485 10.1002/jmri.21085 17968882

[B101] Kaiser T Kellenberger CJ Albisetti M Bergstrasser E Valsangiacomo Buechel ER Normal values for aortic diameters in children and adolescents–assessment in vivo by contrast-enhanced CMR-angiography J Cardiovasc Magn Reson 2008 10 56 10.1186/1532-429X-10-56 19061495 PMC2615773

[B102] Amano Y Takahama K Kumita S Non-contrast-enhanced MR angiography of the thoracic aorta using cardiac and navigator-gated magnetization-prepared three-dimensional steady-state free precession J Magn Reson Imaging 2008 27 3 504 509 10.1002/jmri.21256 18307199

[B103] Potthast S Mitsumori L Stanescu LA Richardson ML Branch K Dubinsky TJ Measuring aortic diameter with different MR techniques: comparison of three-dimensional (3D) navigated steady-state free-precession (SSFP), 3D contrast-enhanced magnetic resonance angiography (CE-MRA), 2D T2 black blood, and 2D cine SSFP J Magn Reson Imaging 2010 31 1 177 184 10.1002/jmri.22016 20027585

[B104] Pepper J Golesworthy T Utley M Chan J Ganeshalingam S Lamperth M Manufacturing and placing a bespoke support for the Marfan aortic root: description of the method and technical results and status at one year for the first ten patients Interact Cardiovasc Thorac Surg 2010 10 3 360 365 10.1510/icvts.2009.220319 20007995

[B105] Han Y Peters DC Salton CJ Bzymek D Nezafat R Goddu B Cardiovascular magnetic resonance characterization of mitral valve prolapse JACC Cardiovasc Imaging 2008 1 3 294 303 10.1016/j.jcmg.2008.01.013 19356441

[B106] Mesana TG Caus T Gaubert J Collart F Ayari R Bartoli J Late complications after prosthetic replacement of the ascending aorta: what did we learn from routine magnetic resonance imaging follow-up? Eur J Cardiothorac Surg 2000 18 3 313 320 10.1016/S1010-7940(00)00512-1 10973541

[B107] Riley P Rooney S Bonser R Guest P Imaging the post-operative thoracic aorta: normal anatomy and pitfalls Br J Radiol 2001 74 888 1150 1158 11777776 10.1259/bjr.74.888.741150

[B108] Albrecht F Eckstein F Matt P Is close radiographic and clinical control after repair of acute type A aortic dissection really necessary for improved long-term survival? Interact Cardiovasc Thorac Surg 2010 11 5 620 625 10.1510/icvts.2010.239764 20709702

[B109] Groenink M de Roos A Mulder BJ Verbeeten B Jr Timmermans J Zwinderman AH Biophysical properties of the normal-sized aorta in patients with Marfan syndrome: evaluation with MR flow mapping Radiology 2001 219 2 535 540 11323484 10.1148/radiology.219.2.r01ma01535

[B110] Adams JN Brooks M Redpath TW Smith FW Dean J Gray J Aortic distensibility and stiffness index measured by magnetic resonance imaging in patients with Marfan’s syndrome Br Heart J 1995 73 3 265 269 10.1136/hrt.73.3.265 7727188 PMC483810

[B111] Baumgartner D Baumgartner C Schermer E Engl G Schweigmann U Matyas G Different patterns of aortic wall elasticity in patients with Marfan syndrome: a noninvasive follow-up study J Thorac Cardiovasc Surg 2006 132 4 811 819 10.1016/j.jtcvs.2006.07.001 17000292

[B112] Lalande A Khau van Kien P Salve N Ben Salem D Legrand L Walker PM Automatic determination of aortic compliance with cine-magnetic resonance imaging: an application of fuzzy logic theory Invest Radiol 2002 37 12 685 691 10.1097/00004424-200212000-00008 12447002

[B113] Metafratzi ZM Efremidis SC Skopelitou AS De Roos A The clinical significance of aortic compliance and its assessment with magnetic resonance imaging J Cardiovasc Magn Reson 2002 4 4 481 491 12549235 10.1081/jcmr-120016386

[B114] Dogui A Redheuil A Lefort M DeCesare A Kachenoura N Herment A Measurement of aortic arch pulse wave velocity in cardiovascular MR: comparison of transit time estimators and description of a new approach J Magn Reson Imaging 2011 33 6 1321 1329 10.1002/jmri.22570 21591000

[B115] Westenberg JJ de Roos A Grotenhuis HB Steendijk P Hendriksen D van den Boogaard PJ Improved aortic pulse wave velocity assessment from multislice two-directional in-plane velocity-encoded magnetic resonance imaging J Magn Reson Imaging 2010 32 5 1086 1094 10.1002/jmri.22359 21031512

[B116] Kroner ES VGR Scholte AJ van den Boogard PJ Hendriksen D Kroft LJ Groenink M Radonic T Bax JJ de Roos A Reiber JH Westenberg JJ Accuracy of aortic pulse wave velcity assessment with velocity-encoded MRI: validation in patients with Marfan syndrome. SCMR/Euro CMR Joint Scientific Sessions 2011 France: Nice 071

[B117] Kroner ES Scholte AJ de Koning PJ van den Boogaard PJ Kroft LJ van der Geest RJ MRI-assessed regional pulse wave velocity for predicting absence of regional aorta luminal growth in marfan syndrome Int J Cardiol 2012 10.1016/j.ijcard.2012.08.057 23000269

[B118] Witte PD RT Laan K Zwinderman AH Mulder BJ Groenink M Aortic distensibility is a predictor for aortic events in patients with marfan syndrome, a 12 year-survival analysis JACC 2010 50 10 0735 1097

[B119] Nollen GJ Groenink M Tijssen JG Van Der Wall EE Mulder BJ Aortic stiffness and diameter predict progressive aortic dilatation in patients with Marfan syndrome Eur Heart J 2004 25 13 1146 52 10.1016/j.ehj.2004.04.033 15231373

[B120] Groenink M de Roos A Mulder BJ Spaan JA van der Wall EE Changes in aortic distensibility and pulse wave velocity assessed with magnetic resonance imaging following beta-blocker therapy in the Marfan syndrome Am J Cardiol 1998 82 2 203 8 10.1016/S0002-9149(98)00315-4 9678292

[B121] Nollen GJ Westerhof BE Groenink M Osnabrugge A van der Wall EE Mulder BJ Aortic pressure-area relation in Marfan patients with and without beta blocking agents: a new non-invasive approach Heart 2004 90 3 314 8 10.1136/hrt.2003.010702 14966057 PMC1768140

[B122] Fattori R Bacchi Reggiani L Pepe G Napoli G Bna C Celletti F Magnetic resonance imaging evaluation of aortic elastic properties as early expression of Marfan syndrome J Cardiovasc Magn Reson 2000 2 4 251 6 10.3109/10976640009148688 11545123

[B123] Eichhorn JG Krissak R Rudiger HJ Ley S Arnold R Boese J Compliance of the normal-sized aorta in adolescents with Marfan syndrome: comparison of MR measurements of aortic distensibility and pulse wave velocity. RoFo Fortschr Geb Rontgenstr Nuklearmed 2007 179 8 841 6 10.1055/s-2007-963192 17638174

[B124] Westenberg JJ SA Vaskova Z Groenink M van der Geest RJ Labadie G van den Boogaard PJ Radonic TR Hilhorst-Hofstee Y Kroft LJ de Roos A Reiber JH Relation between age and aortic wall compliance in the Marfan syndrome: evaulation with Velocity-Encoded MRI. SCMR Scientific Sessions 2010 Pheonix, AZ: BioMed Central 9

[B125] Kvitting JP Ebbers T Wigstrom L Engvall J Olin CL Bolger AF Flow patterns in the aortic root and the aorta studied with time-resolved, 3-dimensional, phase-contrast magnetic resonance imaging: implications for aortic valve-sparing surgery J Thorac Cardiovasc Surg 2004 127 6 1602 7 10.1016/j.jtcvs.2003.10.042 15173713

[B126] Bogren HG Mohiaddin RH Yang GZ Kilner PJ Firmin DN Magnetic resonance velocity vector mapping of blood flow in thoracic aortic aneurysms and grafts J Thorac Cardiovasc Surg 1995 110 3 704 14 10.1016/S0022-5223(95)70102-8 7564437

[B127] Weigang E Kari FA Beyersdorf F Luehr M Etz CD Frydrychowicz A Flow-sensitive four-dimensional magnetic resonance imaging: flow patterns in ascending aortic aneurysms Eur J Cardiothorac Surg 2008 34 1 11 6 10.1016/j.ejcts.2008.03.047 18515137

[B128] Bieging ET Frydrychowicz A Wentland A Landgraf BR Johnson KM Wieben O In vivo three-dimensional MR wall shear stress estimation in ascending aortic dilatation J Magn Reson Imaging 2011 33 3 589 97 10.1002/jmri.22485 21563242 PMC3078726

[B129] Pitcher ACT Suttie J Francis JM Leeson P Blair E Wordsworth BP Forfar JC Markl M Neubauer SN Petersen SE 4D-Flow CMR Demonstrates the Regional Distribution of Aortic Flow Disturbance in Marfan Syndrome Heart 2011 97 Suppl 1 A62

[B130] Pitcher ACT Suttie J Francis JM Leeson P Blair E Wordsworth BP Forfar JC Myerson SG Markl M Neubauer SN Petersen SE Visualisation of aortic flow disturbance in Marfan syndrome by 4D phase-contrast CMR. SCmR/Euro CMR Joint Scientific Sessions 2011 Nice, France: BioMed Central 201

[B131] Camarda JA EM Dholakia RJ Wang H Kwon S LaDisa JF Samyn MM Biophysical properties of the aorta in patients with Marfan syndrome and related connective tissue disorders: evaluation with MRI and computational fluid dynamics modeling. SCMR/Euro CMR Joint Scientific Sessions 2011 Nice, France: BioMed Central 219

[B132] Borghi A Wood NB Mohiaddin RH Xu XY 3D geometric reconstruction of thoracic aortic aneurysms Biomed Eng Online 2006 5 59 10.1186/1475-925X-5-59 17081301 PMC1635716

[B133] Markl M Wallis W Harloff A Reproducibility of flow and wall shear stress analysis using flow-sensitive four-dimensional MRI J Magn Reson Imaging 2011 33 4 988 94 10.1002/jmri.22519 21448968

[B134] Habermann CR Weiss F Schoder V Cramer MC Kemper J Wittkugel O MR evaluation of dural ectasia in Marfan syndrome: reassessment of the established criteria in children, adolescents, and young adults Radiology 2005 234 2 535 41 10.1148/radiol.2342031497 15616116

